# MiRNA-1202 promotes the TGF-β1-induced proliferation, differentiation and collagen production of cardiac fibroblasts by targeting nNOS

**DOI:** 10.1371/journal.pone.0256066

**Published:** 2021-08-24

**Authors:** Jingwen Xiao, Yan Zhang, Yuan Tang, Hengfen Dai, Yu OuYang, Chuanchuan Li, Meiqin Yu

**Affiliations:** 1 The Department of Cardiovascular Medicine, FuZhou First Hospital, FuZhou, Fujian, P.R. China; 2 The Cardiac Function Laboratory of Cardiovascular Medicine, FuZhou First Hospital, FuZhou, Fujian, P.R. China; 3 The Department of Clinical Pharmacy, FuZhou First Hospital, FuZhou, Fujian, P.R. China; The Open University, UNITED KINGDOM

## Abstract

**Background:**

Atrial fibrillation (AF) is a clinically common arrhythmia that affects human health. Myocardial fibrosis serves as an important contributor to AF. Recently, miRNA-1202 have been reported to be up-regulated in AF. However, the role of miRNA-1202 and its mechanism in myocardial fibrosis remain unclear.

**Methods:**

Human cardiac fibroblasts (HCFs) were used to construct a fibrosis model by TGF-β1 induction. The expression of miR-1202 was measured by qRT-PCR. Cell proliferation was assessed by CCK-8 assays. Protein expression levels were measured by western blot. Collagen accumulation was measured by ELISA. The relationship between miR-1202 and nNOS was investigated by luciferase reporter assays.

**Results:**

MiR-1202 expression was obviously increased in HCFs and was both time- and dose-independent. MiR-1202 could increase the proliferation and collagen I, collagen III, and α-SMA levels with or without TGF-β1. MiR-1202 could also increase TGF-β1 and p-Smad2/3 protein levels in comparison to the control group. However, they were obviously decreased after inhibitor transfection. MiR-1202 targets nNOS for negative regulation of HCFs fibrosis by decreasing cell differentiation, collagen deposition and the activity of the TGF-β1/Smad2/3 pathway. Co-transfection of miR-1202 inhibitor and siRNA of nNOS inhibited nNOS protein expression, thereby enhancing the HCFs proliferation. Furthermore, co-transfection of the miR-1202 inhibitor and siRNA of nNOS significantly promoted collagen I, collagen III, TGF-β1, Smad2/3 and α-SMA protein expression and Smad2/3 protein phosphorylation. These findings suggested that miR-1202 promotes HCFs transformation to a pro-fibrotic phenotype by targeting nNOS through activating the TGF-β1/Smad2/3 pathway.

## Introduction

Atrial fibrillation (AF) is the most prevalent sustained arrhythmia in clinical practice, and it is responsible for a high burden of morbidity and mortality due to the limited efficacy of the available therapeutic approaches [1, 2]. Myocardial fibrosis is one of the pathogenesis of atrial fibrillation [3]. Therefore, knowledge of the underlying molecular mechanisms of myocardial fibrosis in AF are required to develop novel therapeutic approaches for AF therapeutic interventions.

Cardiac fibroblasts (CFs) are the major type of cells responsible for myocardial fibrosis [4]. In the cardiac fibroblasts model, excessive deposition of extracellular matrix (ECM) leads to cardiac fibrosis [5]. ECM is essential in maintaining cardiac function and is also an important component in the progression of cardiac fibrosis [6]. A series of studies have demonstrated that transforming growth factor (TGF)-β1 is an important pro-fibrotic growth factor that is involved in the upstream regulation of various biological processes and signaling pathways and directly stimulates cardiac fibroblasts proliferation; the TGF-β1 pathway induces myocardial fibrosis in response to various factors [7]. Ligand binding of TGF-β1 to its type-I and type-II receptors through different pathways, such as Smad, TGF-β protein kinase, and connective tissue growth factor, can increase collagen synthesis and inhibit collagen degradation, resulting in an imbalance in extracellular matrix synthesis and degradation, thereby increasing collagen deposition in the stroma [8–11]. Therefore, cardiac fibroblasts could be used as a cellular model to study myocardial fibrosis induced by TGF-β1.

Previous studies have indicated that 28 miRNAs were differentially expressed in mitral stenosis (MS) patients with atrial fibrillation (AF) compared with the MS patients without AF. Among these, miR-1202 was the most up-regulated [12]. MicroRNAs (miRs) are a class of small, non-coding, endogenous RNAs that consist of 21–25 ribonucleotides and can bind to the 3’-untranslated region (3’UTR) of target mRNAs to regulate gene expression [13–15].

Previous data indicated that miR-939 directly regulates the expression of human inducible nitric oxide synthase (hiNOS) by binding to its 3′-UTR, thereby blocking translation. These results indicated that the regulation of iNOS expression leads to translational inhibition by increasing miR-939 expression [16]. Additionally, other results suggested that atrial-specific up-regulation of miR-31 was a key anti-fibrotic mechanism by causing atrial loss of dystrophin and neuronal NOS (nNOS), which is a key enzyme in the synthesis of nitric oxide (NO) to anti-fibrotic by decreasing the mRNA and protein expression of fiber-junction protein and collagen I and III [17, 18].

Increased nNOS expression in the sarcolemmal membrane and sarcoplasmic reticulum of cardiomyocytes was proven to prevent arrhythmic death after myocardial infarction [19–21]. Furthermore, it was previously reported that NO could induce Smad2/3 protein degradation leading to the inhibition of TGF-β1-induced signaling [22], and it also increased the release of TGF-β1 in myocytes [23]. Thus, nNOS signaling may be essential during the progression of myocardial fibrosis.

Myocardial fibrosis mainly manifests as an imbalance between the synthesis and degradation of extracellular matrix, with increasing collagen deposition in the interstitium, imbalances in collagen composition and arrangement, and remodeling of the extracellular matrix. These effects lead to changes in the electrical coupling and conduction of atrial muscle and the formation of diffuse fibrotic scars. These effects are mainly due to fibroblasts producing collagen and contributing to reactive fibrosis [24]. The principal manifestation of the myocardial fibrosis is pro-fibrogenic transformation of fibroblasts. However, the roles of nNOS-derived NO and the effects of TGF-β1 activation on myocardial fibrosis are still unclear. Therefore, in this study, we tested the hypothesis that miR-1202 contributes to fibrosis. We also demonstrated that miR-1202 targets nNOS to regulate TGF-β1-induced proliferation, differentiation and collagen production of cardiac fibroblasts through the TGF-β1/Smad2/3 pathway. Clarifying the role of miR-1202 in myocardial fibrosis will help us to further understand the mechanism of TGF-β1-induced myocardial fibrosis and will provide new targets for the treatment of AF.

## 2. Materials and methods

### 2.1 Cell culture

Human cardiac fibroblasts (HCFs) were isolated from the atrium of a healthy adult (Lonza, CC-2903). Passage 3–5 HCFs were used for the experiments. The cells were maintained at 37°C in a 5% CO_2_ incubator (SANYO, Osaka, Japan) and cultured in DMEM-F12 medium (Gibco^®^, Grand Island, NY, USA) containing 10% fetal bovine serum (PAN bio-tech, P30-3302). The growth medium was replaced with fresh medium every day until the cells reached confluence.

### 2.2 Total RNA isolated and qPCR analysis

Total RNA was isolated from the cells with RNAiso reagent (TaKaRa Company, Dalian, China) according to the manufacturer’s instructions. Then, 2 μg of RNA per sample was used as input material to obtain miRNAs by using a miRNeasy Mini Kit (Qiagen, Germany), and it was reverse transcribed with a TaqMan MicroRNA RT Kit according to the manufacturer’s protocol as previously described [25]. For qPCR, 2.0 μL of the reverse transcription (RT) product was mixed with 10.0 μL of qPCR SYBR® Green Master Mix (A6001, Promega, USA), 1.0 μL of primers and 7.0 μL of nuclease-free water to a final volume of 20.0 μL. According to the method described in a previous study, the mRNA expression was assayed with an ABI 7500 PCR system (ABI, USA) and calculated by the 2^-△△^Ct method [26]. The qPCR conditions were as follows: 95°C for 5 min, followed by 40 cycles at 95°C for 10 s, 60°C for 30 s, 72°C for 30 s, and 95°C for 15 s. The primers were designed and synthesized by Sangon Bio-tech (Shanghai, China). The following primer sequences were used: miR-1202, CGGTGCCAGCTGCAGTG (F) and AGTGCAGGGTCCGAGGTATT (R); RT: GTCGTATCCAGTGCAGGGTCCGAGGTATTCGCACTGGATACGACCTCCCC; U6, CTCGCTTCGGCAGCACATATACT (F) and ACGCTTCACGAATTTGCGTGTC (R); and RT; AAAATATGGAACGCTTCACGAATTTG. The level of miR-1202 was normalized to the U6 level.

### 2.3 Transfection

The following mimics, inhibitors and siRNAs were designed and constructed by Zolgene Biotechnology Co., Ltd. (Fuzhou, CHINA): miR-1202 mimic (sequence: 5′- GUGCCAGCUGCAGUGGGGGAG-3′); mimic NC (sequence: 5′-UCUACUCUUUCUAGGAGGUUGUGA-3′); miR-1202 inhibitor (sequence: 5′-CUCCCCCACUGCAGCUGGCAC-3′); inhibitor NC (sequence: 5′-UCUACUCUUUCUAGGAGGUUGUGA-3′); si-NC (sense: 5-UUCUCCGAACGUGUCACGUTT-3′ and anti-sense: 5’-ACGUGACACGUUCGGAGAATT-3′); and si-nNOS (sense: 5’- GAUCAAUACUAUUCAUCAATT-3′ and anti-sense: 5’-UUGAUGAAUAGUAUUGAUCTT-3′). All mimics, inhibitors or siRNAs were transfected into cells with Lipofectamine™ 2000 transfection reagent (11668027, Invitrogen). The method of transfection followed the manufacturer’s protocol. The mimics final concentration was 20 nM, the inhibitor was 40 nM and the siRNA was 50 nM. The transfection efficiencies after at least 48 h of all of the mimics and inhibitors were verified when the cells were used for further experimentation.

### 2.4 CCK-8 assay

The cell viability was measured by the CCK-8 method using the Cell Counting Kit-8 kit (CK04, Dojindo). The cells were adjusted to 4×10^4^ cells/mL in 96-well plates and incubated for 24 h at 37°C and 5% CO_2_. Then, 100 ng/ml IL-6 was added into the cell plates. After incubating for 24 h at 5% CO_2_ and 37°C with IL-6, 10 μL CCK-8 was added into every well. After 4 h, the values were detected at 450 nm using a Biotek Synergy 2 plate reader (Winooski, VT).

### 2.5 Luciferase reporter assays

The Light switch luciferase assay reagent was obtained from Promega (Corporation, USA). The NOS1-WT and NOS1-MUT were prepared and transfected together with the Renilla luciferase expressing vector and the luciferase reporter plasmid for 24 h. The activity of luciferase was analyzed by a dual reporter assay system according to the manufacturer’s instructions. Renilla luciferase was used for normalization.

### 2.6 Western blot analysis

Total proteins were isolated from the cells, which were collected and mixed with RIPA lysis buffer containing PMSF. The samples were placed on ice for 20 min at 4°C and were then centrifuged for 20 min at 12000 rpm. The supernatant was collected and used to determine the protein concentrations by a BCA protein assay kit PC0020-500 (Solaibao, Beijing, China). Using methods described in a previous study, the proteins were separated on 10–20% SDS-PAGE [27]. After transfer of the separated proteins to an NC membrane, the blots were blocked and washed with TBS containing 0.5% Tween-20 (Beyotime Institute of Biotechnology, Jiangsu, China). The membranes were blocked with 5% bovine serum albumin (BSA) for 1 h at room temperature. After washing three times with TBS, the membranes were incubated with anti-collagen I mAb (Abcam, ab34710, 1:1500 dilution), anti-collagen III mAb (Abcam, ab6310, 1:1500 dilution), anti-alpha smooth muscle actin (Abcam, ab5694, 1:1500 dilution), anti-TGF beta 1 mAb (1:1000 dilution), anti-Smad2/Smad3 mAb (Abcam, ab272332, 1:1500 dilution), anti-p-Smad2/3 mAb (Abcam, ab272332, 1:1500 dilution), anti-TNF-β1 mAb (Abcam, ab215715, 1:1500 dilution), and anti-nNOS mAb (Abcam, ab5586, 1:1500 dilution) overnight at 4°C. The membranes were then incubated with anti-mouse HRP-conjugated secondary antibody (Abcam, 1:5000 dilution) for 2 h at room temperature. Then, the signal was detected by chemiluminescence with Thermo ECL (34080, Thermo). Anti-β-actin (1:5000 dilution) was used as a loading control. All antibodies were purchased from Abcam (Beijing, China). The results were collected by a Versa DocTM imaging system (Peiqing Technology Co. Ltd., Shanghai, China) and analyzed with ImageJ software.

### 2.7 Enzyme-Linked Immunosorbent Assay (ELISA)

Collagen I and Collagen III ELISA kits were purchased from Nanjing Jiancheng Bioengineering Institute (Nanjing, China). The level of collagen I and collagen III in the HCFs culture media were determined by ELISA kits according to the manufacturer’s protocols.

### 2.8 Statistical analysis

One-way ANOVA was used to analyze the results with SPSS 22.0 statistical software. P < 0.05 was marked “*” to show significance. All data are expressed as the mean ± SD.

## 3. Results

### 3.1 miR-1202 expression levels in TGF-β1-induced HCFs

**Fig 1:**
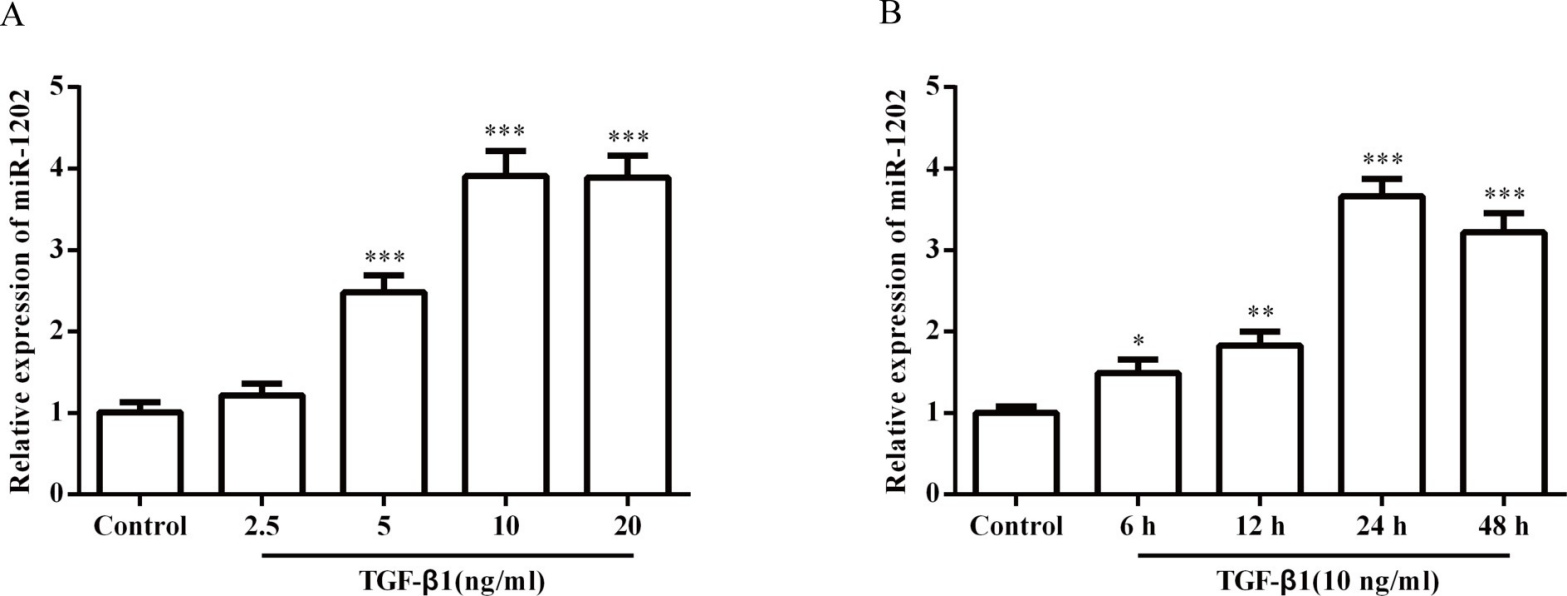
The miR-1202 expression levels in HCFs. (A) The miR-1202 expression level was dose-dependent. (B) The miR-1202 expression level was time-dependent. All of the data are shown as the mean ± SD. Compared with the control group, n = 3, *P < 0.05; **P < 0.01, ***P < 0.001.

For miR-1202 expression in HCFs, both time and dose were evaluated, and the results are shown in Fig 1. miR-1202 expression in HCFs was obviously increased in the groups treated with 5, 10 and 20 ng/mL TGF-β1 (P<0.01 or P<0.001), showing dose dependence. Moreover, the increasing trend tended to be stable between the 10 and 20 ng/mL TGF-β1 treatment groups. In addition, the miR-1202 expression was significantly up-regulated (P<0.05, P<0.01 or P<0.001) in the 10 ng/mL TGF-β1 treatment group, showing time dependence. In the 24-h group, the miR-1202 expression was the highest. Note that the small sample size in this study limits statistical power, so small differences may not have been detected.

### 3.2 Effects of miR-1202 on the TGF-β1-induced proliferation, differentiation, and collagen synthesis of HCFs

**Fig 2:**
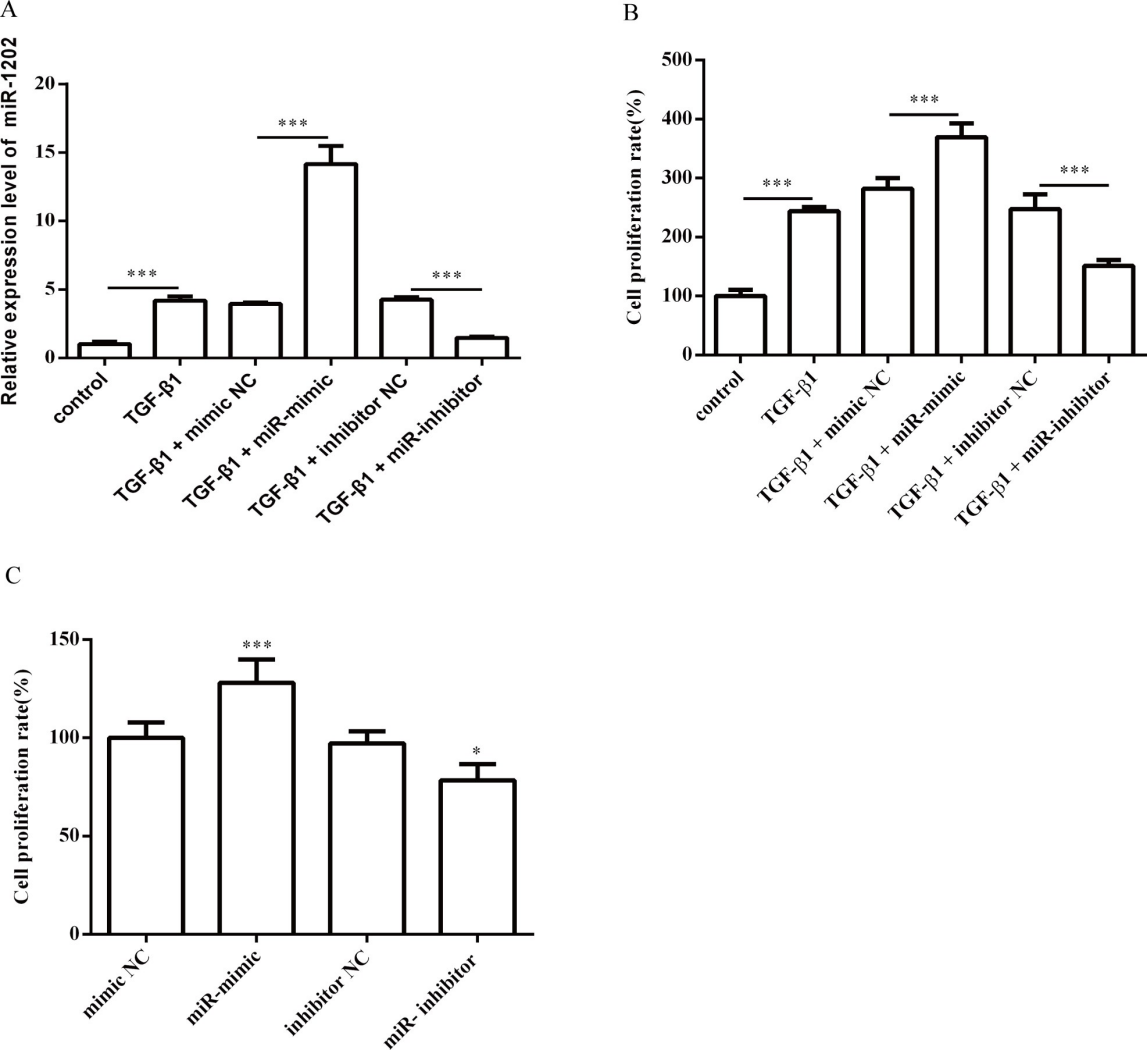
Effects of miR-1202 on the proliferation of HCFs treated with or without TGF-β1. (A) The miR-1202 levels in TGF-β1-induced HCFs cells; (B) The cell viability in TGF-β1-induced HCFs; (C) The cell viability in HCFs without TGF-β1 treatment. All of the data are expressed as the mean ± SD. Compared with the control group, n = 3, *P < 0.05; **P < 0.01, ***P < 0.001. The analysis is not able to detect small significant differences due to small sample size.

**Fig 3:**
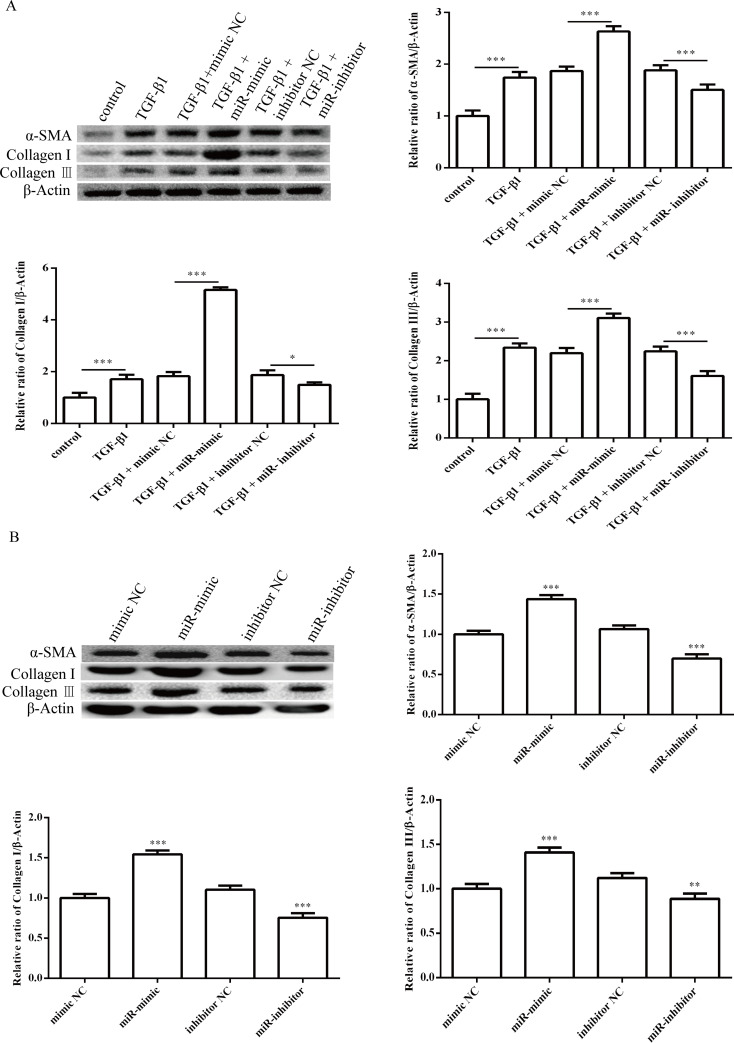
Effects of miR-1202 on the differentiation and collagen synthesis of HCFs treated with or without TGF-β1. The protein expression levels of α-SMA, collagen I and collagen III in HCFs treated with TGF-β1 (A) or in HCFs without TGF-β1 (B) were assessed by western blotting. All of the data are expressed as the mean ± SD. Compared with the control group, n = 3, *P < 0.05; **P < 0.01, ***P < 0.001. The analysis is not able to detect small significant differences due to small sample size.

**Fig 4:**
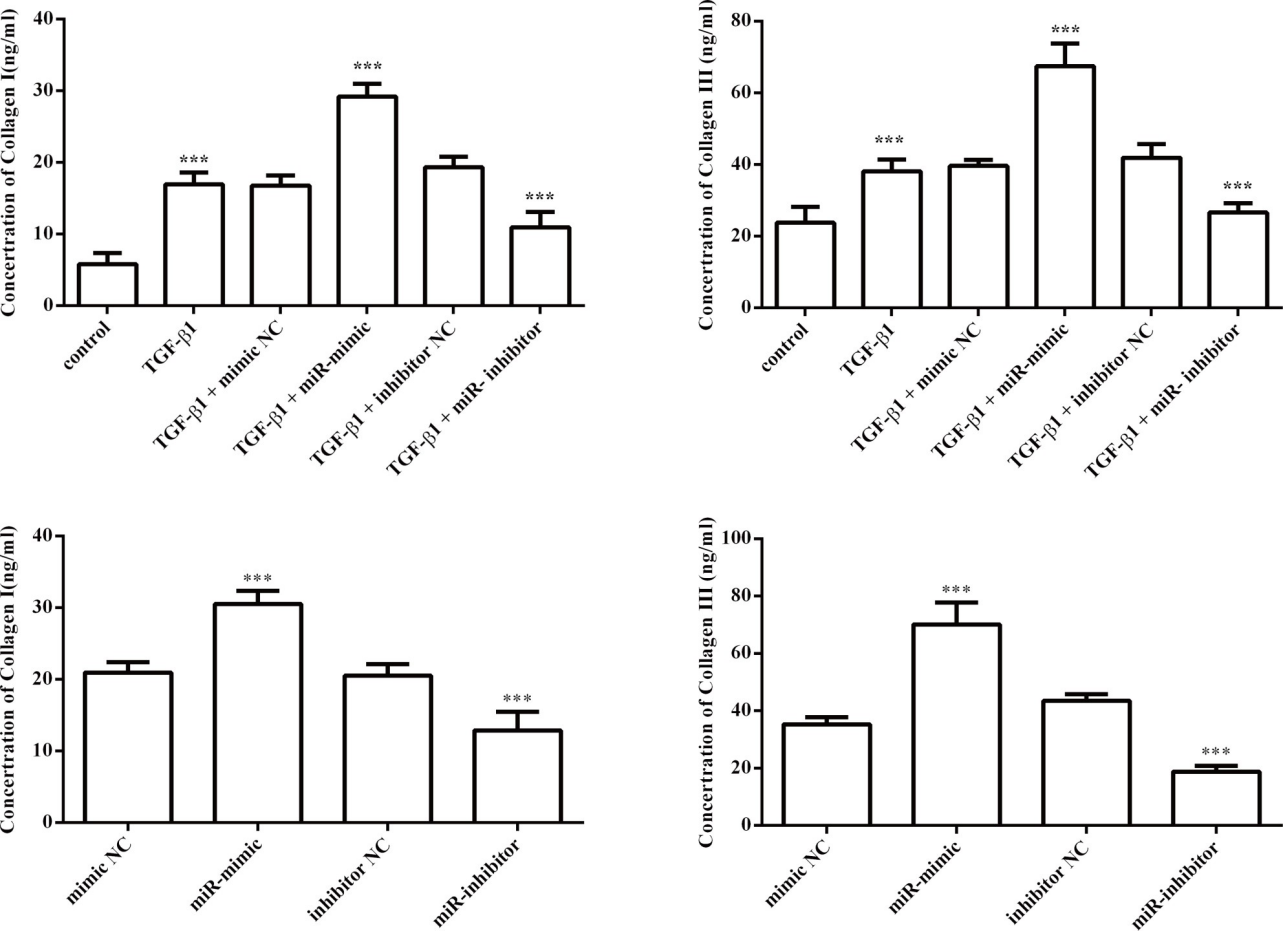
Effects of miR-1202 on collagen synthesis of HCFs treated with or without TGF-β1. The expression levels of collagen I and collagen III in HCFs treated with TGF-β1 (A) or in HCFs without TGF-β1 (B) were assessed by ELISA assay. All of the data are expressed as the mean ± SD. Compared with the control group, n = 3, *P < 0.05; **P < 0.01, ***P < 0.001. The analysis is not able to detect small significant differences due to small sample size.

**Fig 5:**
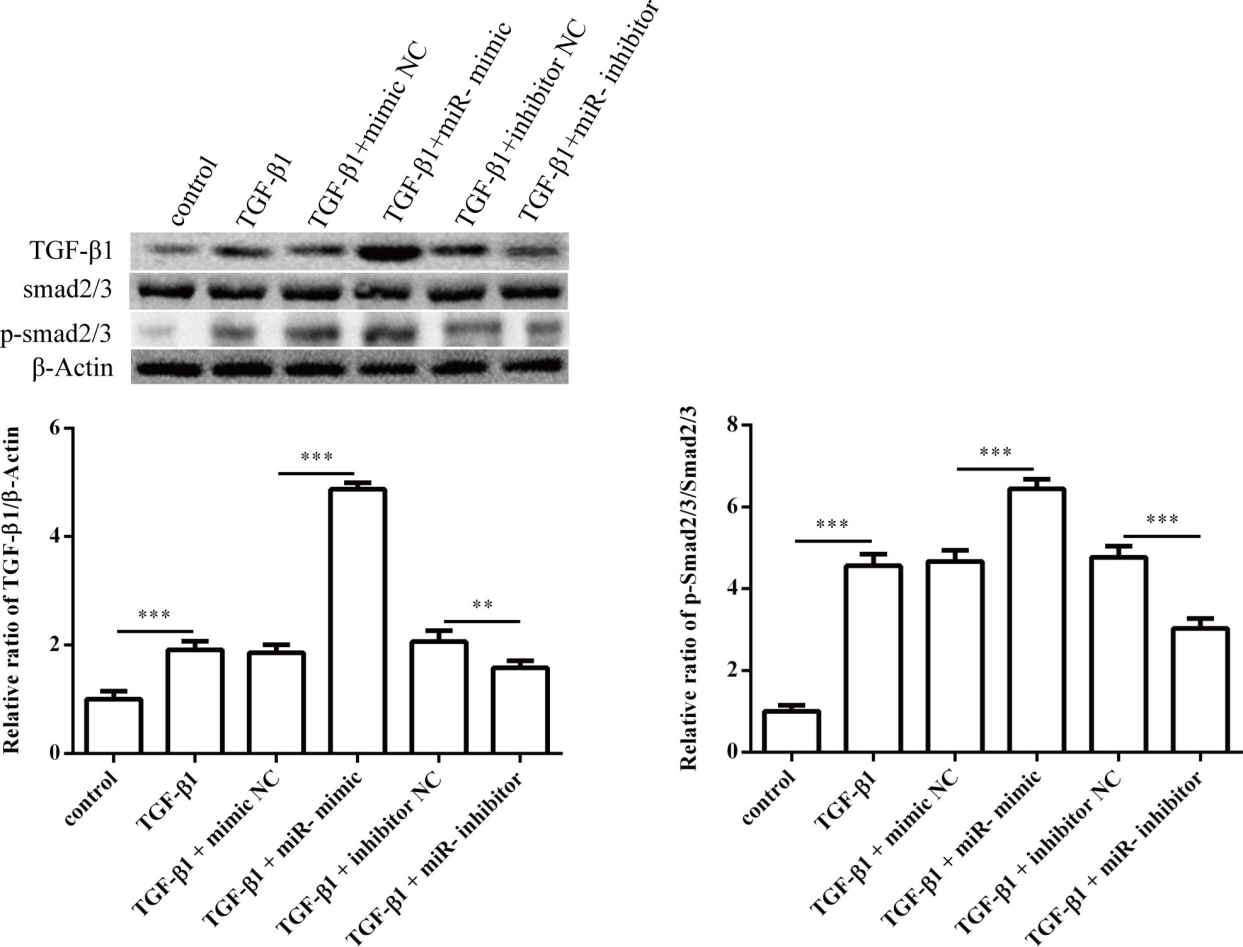
Effects of miR-1202 on the TGF-β1/Smad2/3 pathway. All of the data are shown as the mean ± SD. Compared with the control group. n = 3, *P < 0.05; **P < 0.01; ***P < 0.001. The analysis is not able to detect small significant differences due to small sample size.

Next, we investigated the effect of miR-1202 on the proliferation, differentiation, collagen synthesis and TGF-β1/Smad2/3 pathway in HCFs. The miR-1202 mimic or inhibitor was transfected into HCFs. The transfection effects were confirmed by qRT-PCR. As expected, miR-1202 was up-regulated in the miR-1202 mimic group but was decreased in the knockdown group (Fig 2A). First, we observed that the miR-1202 mimic significantly promoted HCF proliferation with or without TGF-β1 compared with that of control group, while inhibiting miR-1202 suppressed the proliferation of HCFs (Fig 2B and 2C). In addition, the protein level of α-SMA (Fig 3A and 3B), a reliable and classic myofibroblast marker, and the protein levels of collagen I and collagen III (Figs 3A, 3B and 4) in HCFs treated with the miR-1202 mimic were significantly increased with or without TGF-β1 compared with those of the control group, while the miR-1202 inhibitor inhibited the expression of α-SMA (Fig 3A and 3B), collagen I and collagen III (Figs 3A, 3B and 4) with or without TGF-β1. Furthermore, our results demonstrated that the miR-1202 mimic increased the protein expression levels of TGF-β1 and p-Smad2/3 in TGF-β1-treated HCFs; however, the miR-1202 inhibitor showed the opposite effects (Fig 5). Collectively, these data indicated that miR-1202 promotes HCFs proliferation, differentiation, and collagen synthesis, and activates the TGF-β1/Smad2/3 pathway in HCFs. Note that the small sample size in this study limits statistical power, so small differences may not have been detected.

### 3.3 Effect of miR-1202 on TGF-β1 induced proliferation, differentiation and collagen synthesis of HCFs by targeting nNOS

**Fig 6:**
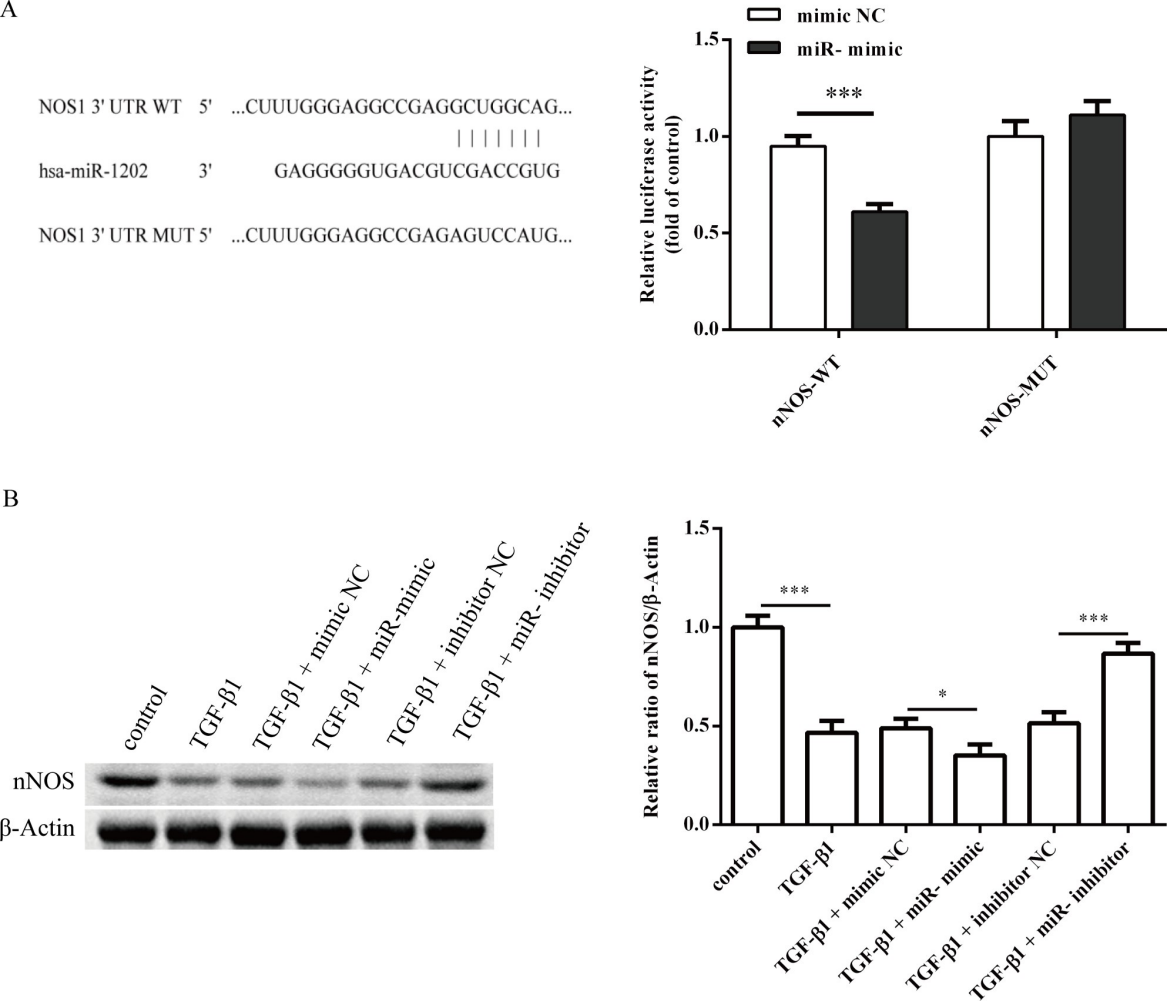
nNOS is a target of miR-1202. (A) Left: Binding sites for miR-1202 in the 3’-UTR of nNOS mRNA; Right: Luciferase reporter assay showing reduced luciferase reporter activity in HCFs containing the nNOS-WT 3’-UTR fragment. (B) nNOS protein expression in HCFs infected with miR-1202 mimic or miR-1202 inhibitor was measured after 48 h. All of the data are expressed as the mean ± SD. Compared with the control group. n = 3, *P < 0.05; **P < 0.01; ***P < 0.001. The analysis is not able to detect small significant differences due to small sample size.

To analyze miR-1202 as a candidate to regulate pro-fibrotic activities, it was searched using Target-Scans (http://www.targetscan.org/). Luciferase reporter assays containing the wild-type (wt) or mutant (mut) miR-1202 target sites in the NOS1 3′-UTR were performed in HCFs. The results showed that the miR-1202 mimic obviously reduced the luciferase activity of the NOS1 WT 3′-UTR relative to that of the mimic control group (P<0.01), but the NOS1 MUT 3′-UTR showed no significant effect (Fig 6A). In addition, nNOS protein expression in HCFs was also inhibited by miR-1202 mimic transfection and TGF-β1 treatment, but this trend was reversed when the miR-1202 inhibitor was transfected (Fig 6B). These results indicated that miR-1202 reduced nNOS expression by binding to target sites in its mRNA. Note that the small sample size in this study limits statistical power, so small differences may not have been detected.

**Fig 7:**
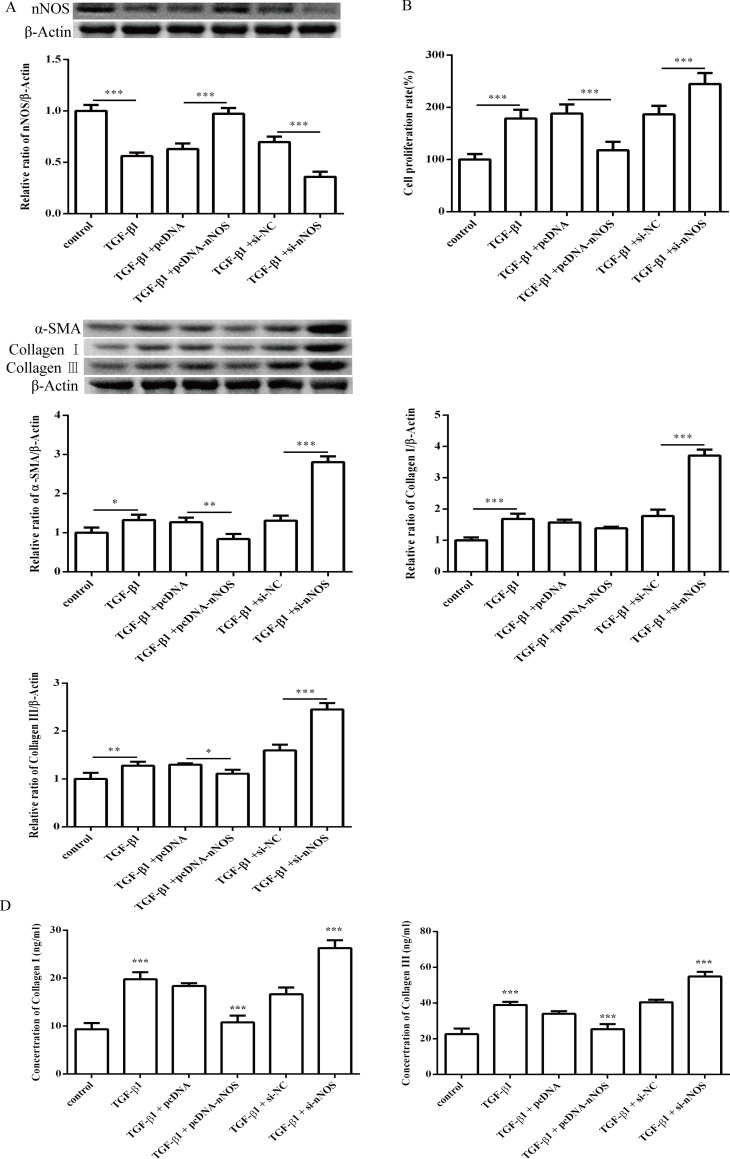
Effect of nNOS on TGF-β1 induced proliferation, differentiation and collagen synthesis of HCFs. (A) The nNOS levels in TGF-β1-induced HCFs; (B) The cell viability of TGF-β1-induced HCFs; (C) The protein expression levels of α-SMA, collagen I and collagen III in TGF-β1-treated HCFs were assessed by western blotting; (D) The expression levels of collagen I and collagen III in TGF-β1-treated HCFs were assessed by ELISA assay. All of the data are shown as the mean ± SD. Compared with the control group. n = 3, *P < 0.05; **P < 0.01; ***P < 0.001. The analysis is not able to detect small significant differences due to small sample size.

**Fig 8:**
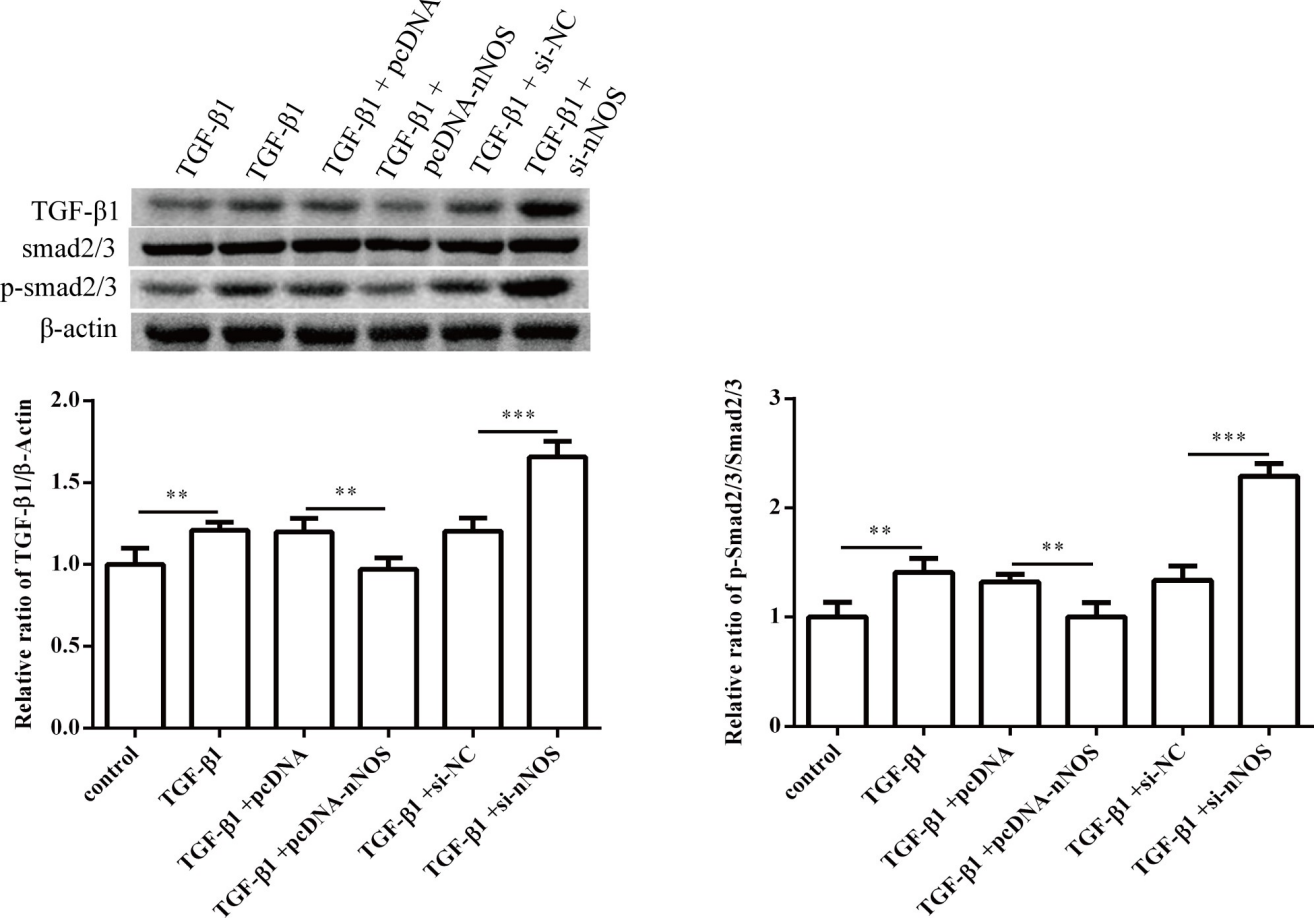
Effects of nNOS on the TGF-β1/Smad2/3 pathway. All of the data are shown as the mean ± SD. Compared with the control group. n = 3, *P < 0.05; **P < 0.01; ***P < 0.001. The analysis is not able to detect small significant differences due to small sample size.

To evaluate the effect of nNOS expression changes on myocardial fibrosis, HCFs were transfected with pcDNA-nNOS or si-nNOS. Western blot analysis showed that the nNOS protein level was increased in HCFs transfected with pcDNA-nNOS and significantly decreased in HCFs transfected with si-nNOS (Fig 7A). The viability of HCFs was significantly reduced after nNOS over-expression, but it was enhanced by transfection of si-nNOS (Fig 7B). Over-expression of nNOS markedly inhibited the increase in the TGF-β1-induced levels of α-SMA, collagen I and collagen III (Fig 7C and 7D). In contrast, knockdown of nNOS promoted TGF-β1-stimulated expression of α-SMA, collagen I and collagen III (Fig 7C and 7D). Furthermore, our results demonstrated that pcDNA-nNOS decreased the protein expression levels of TGF-β1 and p-Smad2/3 in TGF-β1-treated HCFs, however, si-nNOS showed the opposite results (Fig 8). These findings indicated that nNOS protected HCFs from TGF-β1-induced differentiation and collagen synthesis through the TGF-β1/Smad2/3 pathway. Note that the small sample size in this study limits statistical power, so small differences may not have been detected.

### 3.4 Effect of miR-1202 and nNOS co-transfection on TGF-β1 induced proliferation, differentiation and collagen synthesis of HCFs

**Fig 9:**
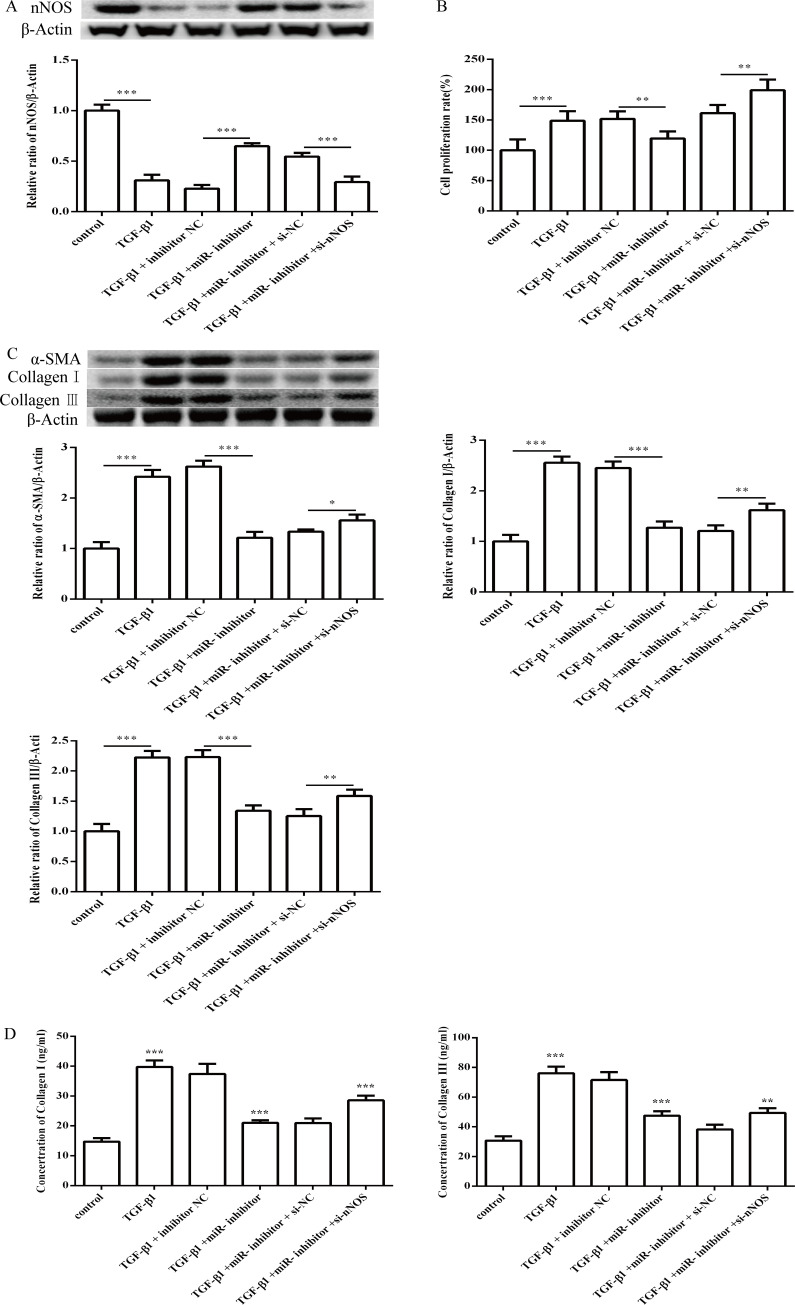
Effect of miR-1202 and nNOS co-transfection on TGF-β1 induced proliferation, differentiation and collagen synthesis of HCFs. (A) nNOS protein expression in TGF-β1-induced HCFs; (B) The cell viability in TGF-β1-induced HCFs; (C) The protein expression levels of α-SMA, collagen I and collagen III in TGF-β1-treated HCFs were assessed by western blotting; (D) The expression levels of collagen I and collagen III in TGF-β1-treated HCFs were assessed by ELISA assay. All of the data are expressed as the mean ± SD. Compared with the control group. n = 3, *P < 0.05; **P < 0.01; ***P < 0.001. The analysis is not able to detect small significant differences due to small sample size.

**Fig 10:**
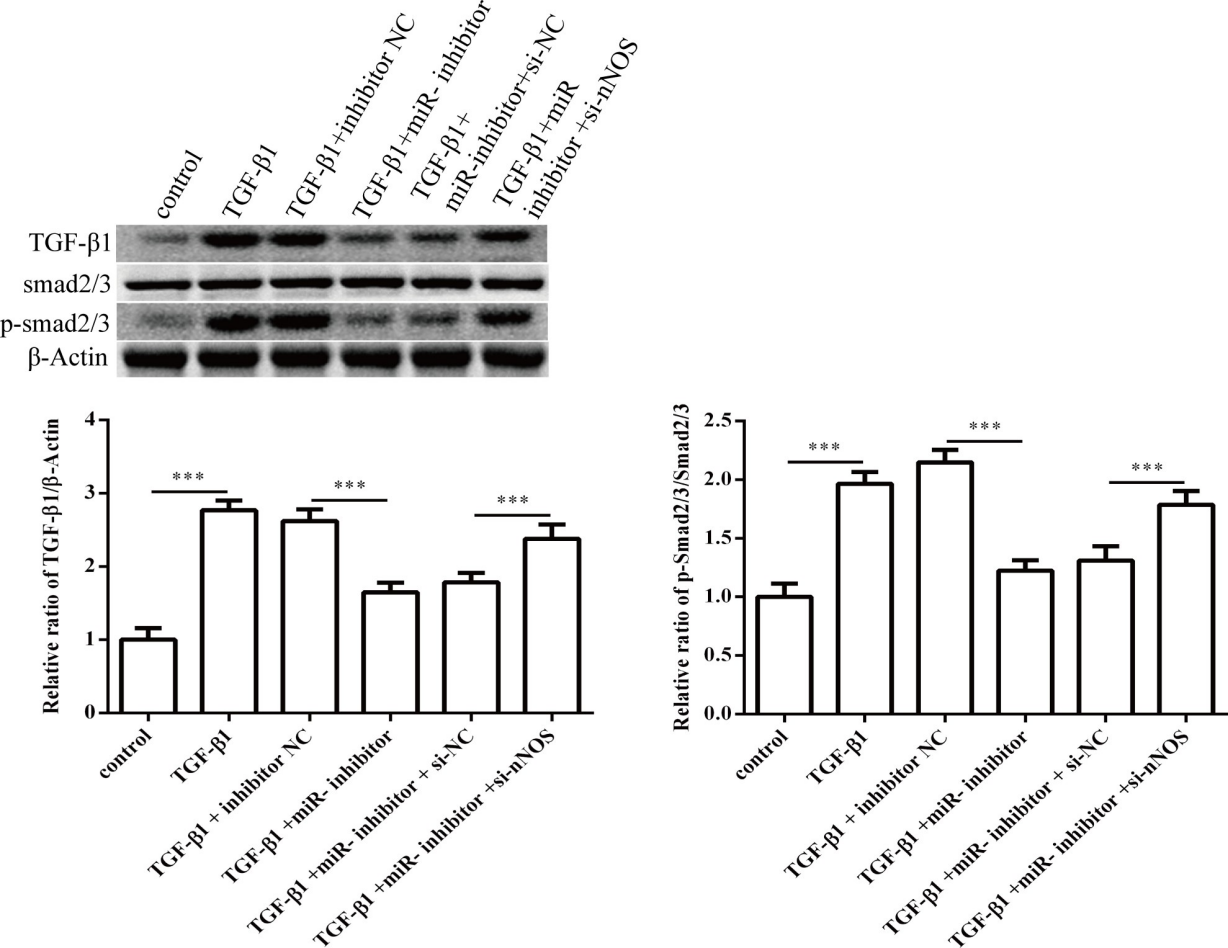
Effect of miR-1202 and nNOS co-transfection on the TGF-β1/Smad2/3 pathway. All of the data are expressed as the mean ± SD. Compared with the control group. n = 3, *P < 0.05; **P < 0.01; ***P < 0.001. The analysis is not able to detect small significant differences due to small sample size.

To determine the role of nNOS in miR-1202 regulation of the TGF-β1-induced proliferation, differentiation and collagen synthesis of HCFs, we co-transfected the miR-1202 inhibitor and si-nNOS into TGF-β1-induced HCFs. The results showed that the nNOS protein level was significantly enhanced after the miR-1202 inhibitor transfection (P<0.01), but it was obviously inhibited (P<0.01) with the co-transfection of the miR-1202 inhibitor and si-nNOS (Fig 9A). The viability of HCFs was significantly inhibited (P<0.01) after the miR-1202 inhibitor transfection in comparison to the only TGF-β1 treated group, but the effect was obviously enhanced (P<0.05) when the cells were co-transfected with the miR-1202 inhibitor and si-nNOS (Fig 9B). These results revealed that co-transfection of the miR-1202 inhibitor and si-nNOS significantly promoted collagen I, collagen III and α-SMA protein expression (Fig 9C) and increased the TGF-β1 and Smad2/3 protein expression and Smad2/3 protein phosphorylation (Fig 10). These results showed that miR-1202 targets nNOS to inhibit nNOS expression and negatively regulated HCFs pro-fibrotic activities through the TGF-β1/Smad2/3 pathway. Note that the small sample size in this study limits statistical power, so small differences may not have been detected.

## Discussion

AF is one of the most common arrhythmias, which places a huge burden on society, but its pathogenic mechanism remains largely unknown. Myocardial fibrosis induced by pro-fibrotic responses is thought to significantly affect the development of AF [4]. In the present study, miR-1202 levels were obviously increased in the TGF-β1-treated HCF in a time- and dose-dependent fashion. Additionally, transfection of the miR-1202 mimic significantly increased the HCF proliferation. This is consistent with previous research, which described that TGF-β1 over-expression could enhance the pro-fibrotic remodeling of the myocardium in a mouse model [28]. In the present study, the results indicated that miR-1202 mimic transfection obviously increased the levels of collagen I, collagen III, α-SMA, and Smad2/3 protein and Smad2/3 protein phosphorylation, which could increase the deposition of ECM.

In addition, the miR-1202 promoted TGF-β1-induced pro-fibrotic responses in HCFs possibly by enhancing collagen deposition and activation of the TGF-β1/Smad2/3 pathway. Relevant studies have suggested that in the heart, Smad2 binds to TGF-β1 and then binds to Smad3 and Smad4 through phosphorylation to form a transcription complex, which is then transferred into the nucleus where it binds to binding elements on DNA, ultimately altering the transcription of target genes [29].

MiR-1202 in HCFs was evaluated as a candidate to regulate pro-fibrotic activities by a luciferase reporter assay with miR-1202 target sites in the NOS1 3′-UTR. The results indicated that miR-1202 reduced nNOS expression by binding to target sites. nNOS is expressed in the sarcolemmal membrane and sarcoplasmic reticulum of myocardial cells and produces NO to regulate sarcolemmal ion conductance and prevent arrhythmic death in mice [17, 20, 21]. Previous results suggested that NO could induce the degradation of Smad2/3, resulting in the suppression of TGF-β1-induced signaling in cultured myocytes [22, 23]. Thus, nNOS signaling may be important in maintaining the function of cardiomyocytes. In the present study, the viability of HCFs was significantly reduced after nNOS over-expression, and it was enhanced by transfection of si-nNOS. These results indicated that over-expressing the nNOS protein could reduce pro-fibrotic effects.

To address the mechanism by which nNOS inhibits HCFs pro-fibrotic phenotypes, the present study revealed that nNOS was repressed by transfecting the siRNA. This significantly promoted collagen and α-SMA protein expression, enhanced TGF-β1 and Smad2/3 protein expression, and increased Smad2/3 protein phosphorylation, thereby activating the TGF-β1/Smad2/3 pathway and enhancing pro-fibrotic responses. This finding indicated that nNOS could be a biomarker for the negative regulation of HCFs pro-fibrotic phenotypes associated with collagen deposition and the TGF-β1/Smad2/3 pathway. A previous study has indicated that TGF-β1 could induce extracellular matrix protein synthesis and TIMP up-regulation through activation of the Smad signal pathway and induce matrix metalloproteinase (MMP) 2 synthesis in myocardial fibroblasts, leading to cardiac remodeling [30]. In addition, TGF-β1 mediates the expression of platelet-derived growth factor D, which also induces the synthesis of MMP-1, 2 and 9 in myocardial fibroblasts [31–33]. As a downstream signaling pathway, TGF-β1 is activated by the Smad pathway, causing cell proliferation, differentiation, transformation and apoptosis; these processes jointly induce myocardial fibroblast differentiation and they participate in the regulation of fibrosis [34]. Our findings indicated that co-transfection of the miR-1202 inhibitor and siRNA of nNOS could repress nNOS protein expression, thereby significantly enhancing HCFs proliferation by promoting collagen I, III and α-SMA protein expression. This subsequently increased the protein levels of TGF-β1 and p-Smad2/3 in TGF-β1 induced pro-fibrotic responses in the HCFs. MiR-1202 targets nNOS, which participates in the regulation of collagen deposition and the TGF-β1/Smad2/3 pathway in pro-fibrotic process, and it also directly stimulates the proliferation of HCFs. Collagen synthesis induced by the TGF-β1/Smad3 pathway promotes pro-fibrotic activation of myocardial fibroblasts [35]. Inhibition of TGF-β1 expression in animal models and in vitro culture can prevent pro-fibrotic responses [36].

In addition, the miR-1202 is noted to be associated with ventricular assist device (VAD) failure [37]. Cardiac fibrosis induced by pro-fibrotic signals was considered as an important pathophysiological contributor to atrial fibrillation [1]. In our study, miR-1202 mediates pro-fibrotic responses in cultured cardiac fibroblasts, indicating that miR-1202 may be a potential trigger of atrial fibrillation. Development of atrial fibrillation episodes is associated with right ventricular failure and increased mortality after ventricular assist devices implantation [38–40]. Therefore, miR-1202 might be up-regulated in the context of VAD failure, and associated with VAD failure may be due to increased atrial fibrillation.

In conclusion, the current study showed that miR-1202 directly targets nNOS and negatively regulates its expression. MiR-1202 is a potential bio-marker that promoted pro-fibrotic activation of HCFs by enhancing collagen deposition and activating the TGF-β1/Smad2/3 pathway. Studying miRNA targeting of the nNOS-mediated TGF-β1/Smad2/3 pathway allows us to better understand the role of miR-1202 in the pathophysiological processes of disease in order to develop therapeutic pathways with higher specificity and fewer side effects. However, we should note that there are some limitations in this study. First, MiR-1202 and miR-31 target 3’UTR of nNOS in different position, and the mechanisms merit further investigation. Second, this study has a small sample size, it associated with a small statistical power.

## Supporting information

S1 Data (ZIP)

S2 Data (ZIP)
