# Supplementary figures and images for: MiRNA-1202 promotes the TGF-β1-induced proliferation, differentiation and collagen production of cardiac fibroblasts by targeting nNOS
Source: PLoS One. 2021 Aug 24;16(8):e0256066. doi: 10.1371/journal.pone.0256066 (PMC8384215; doi:10.1371/journal.pone.0256066)

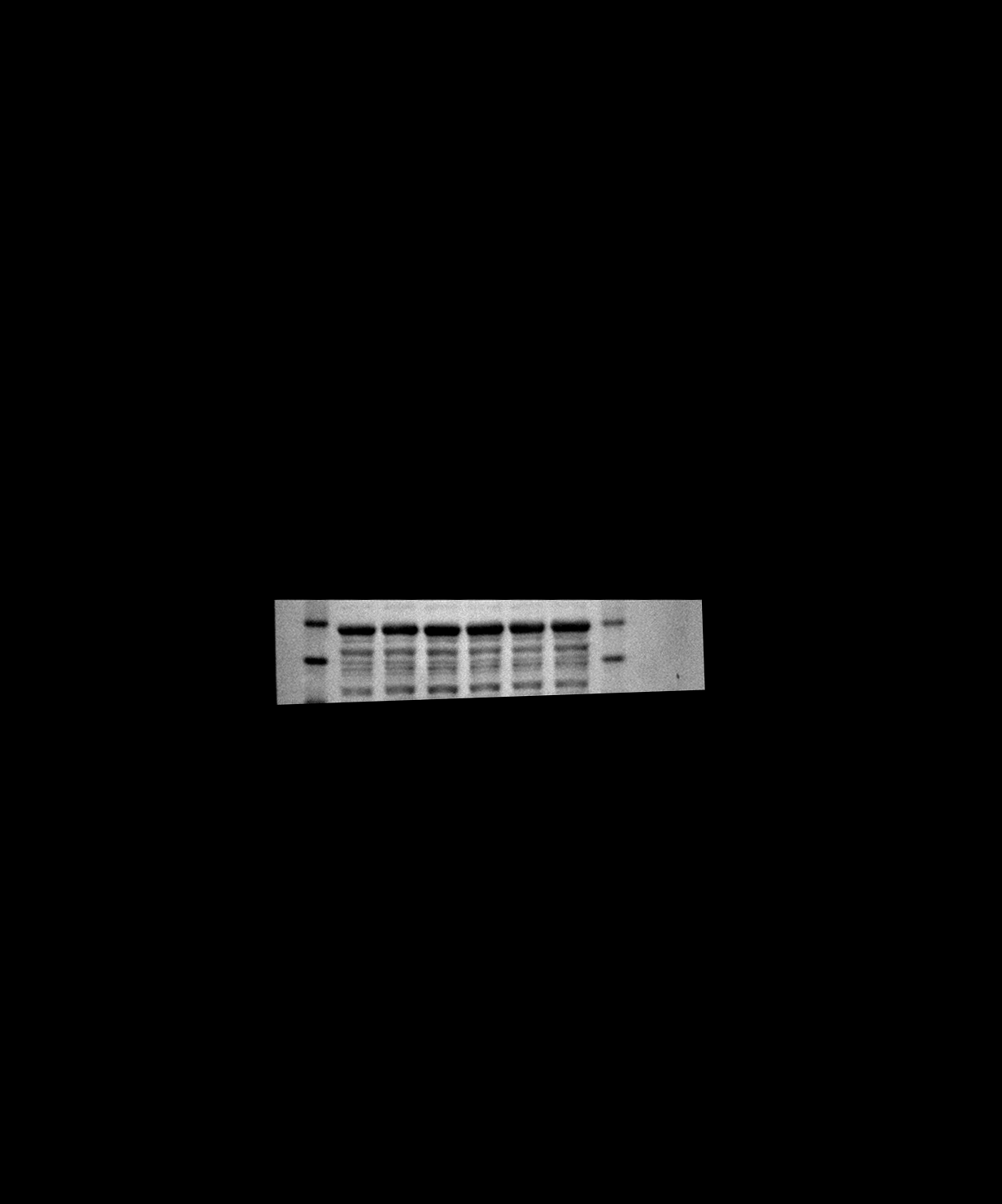

Supplement: S2 Data — (ZIP) [file pone.0256066.s002.zip › WB blots/WB Fig 6/actin.tif]

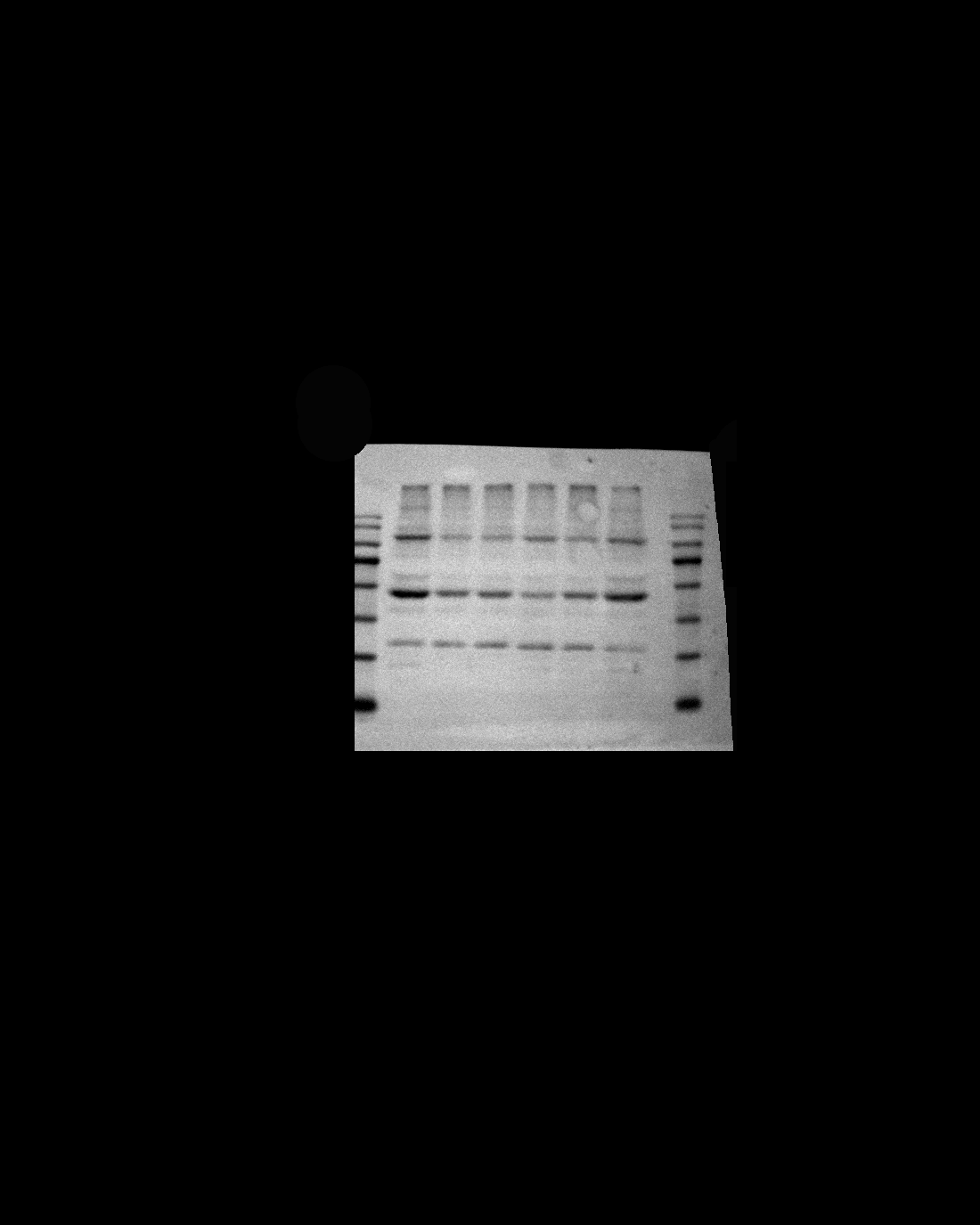

Supplement: S2 Data — (ZIP) [file pone.0256066.s002.zip › WB blots/WB Fig 6/nNOS.tif]

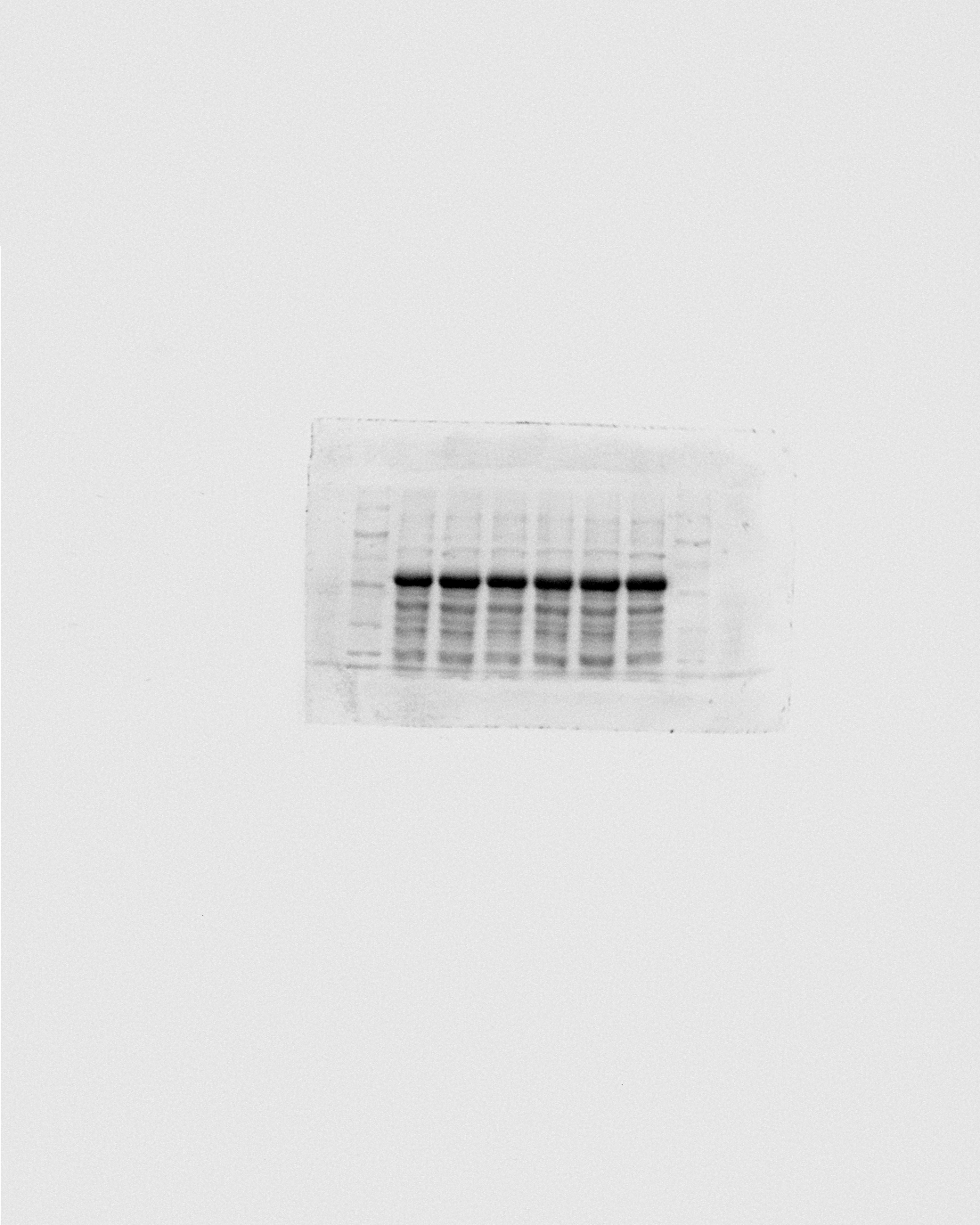

Supplement: S2 Data — (ZIP) [file pone.0256066.s002.zip › WB blots/WB Fig 7/actin-Fig 7A.tif]

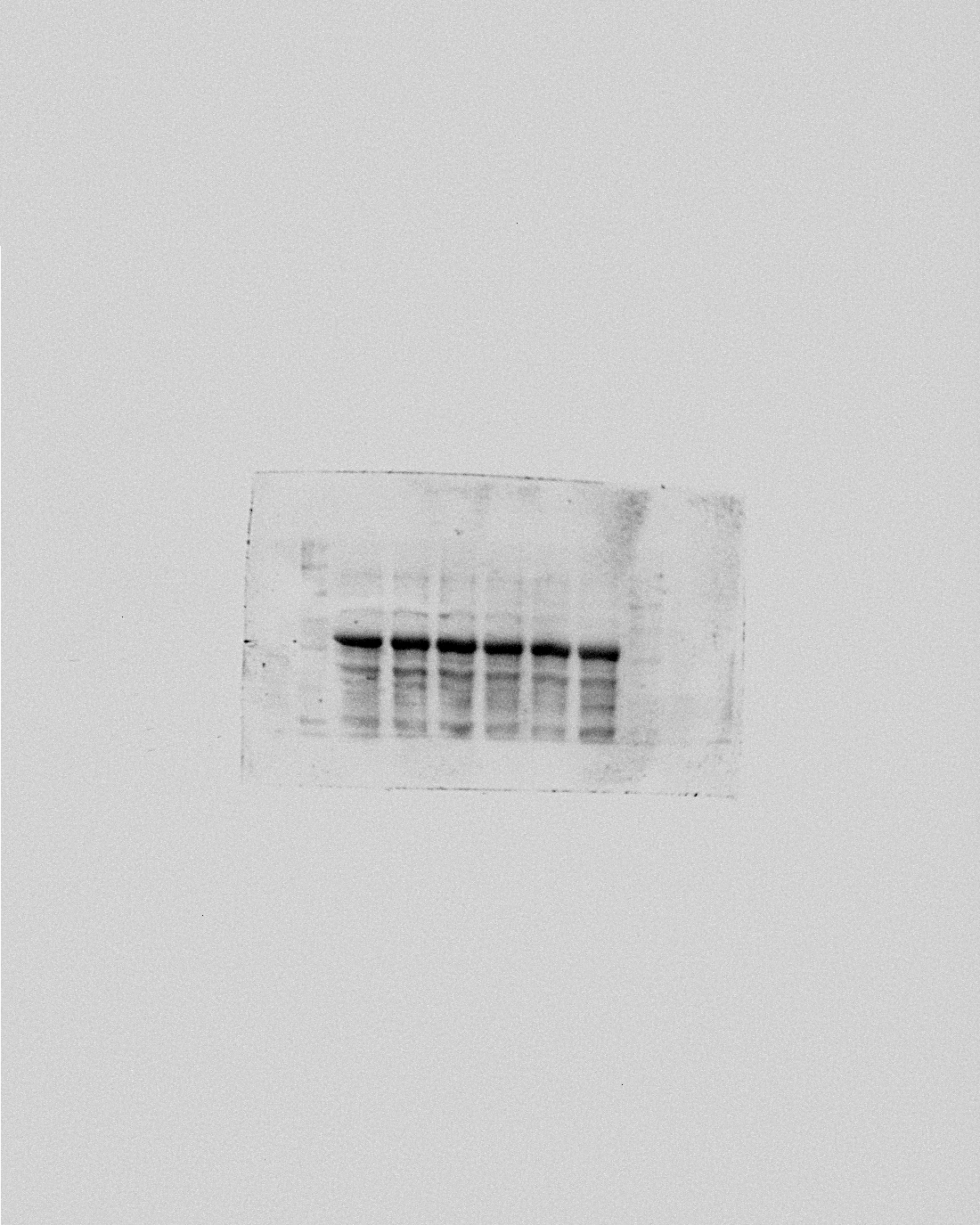

Supplement: S2 Data — (ZIP) [file pone.0256066.s002.zip › WB blots/WB Fig 7/actin-Fig 7C.tif]

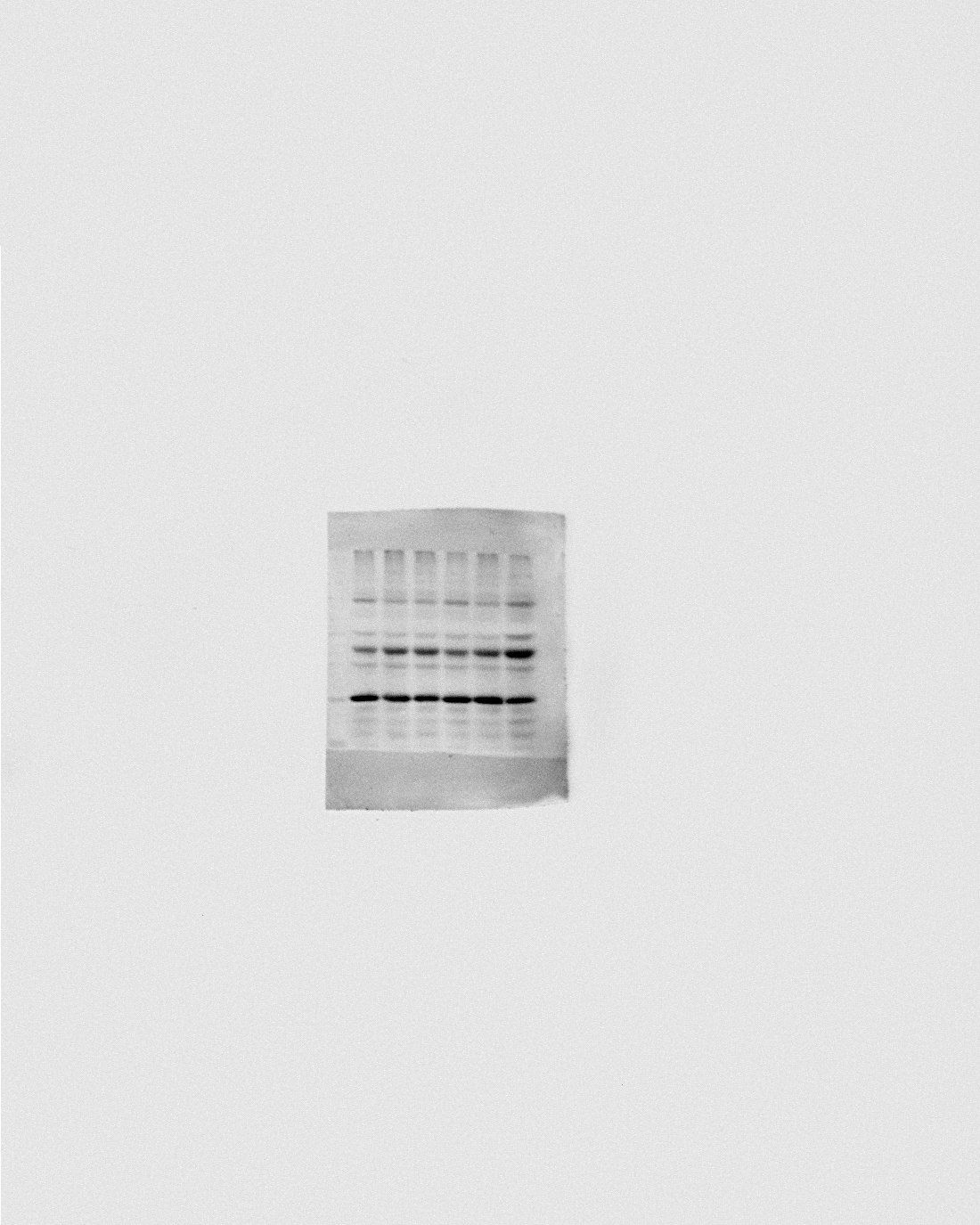

Supplement: S2 Data — (ZIP) [file pone.0256066.s002.zip › WB blots/WB Fig 7/Collagen ó±-Fig 7C.tif]

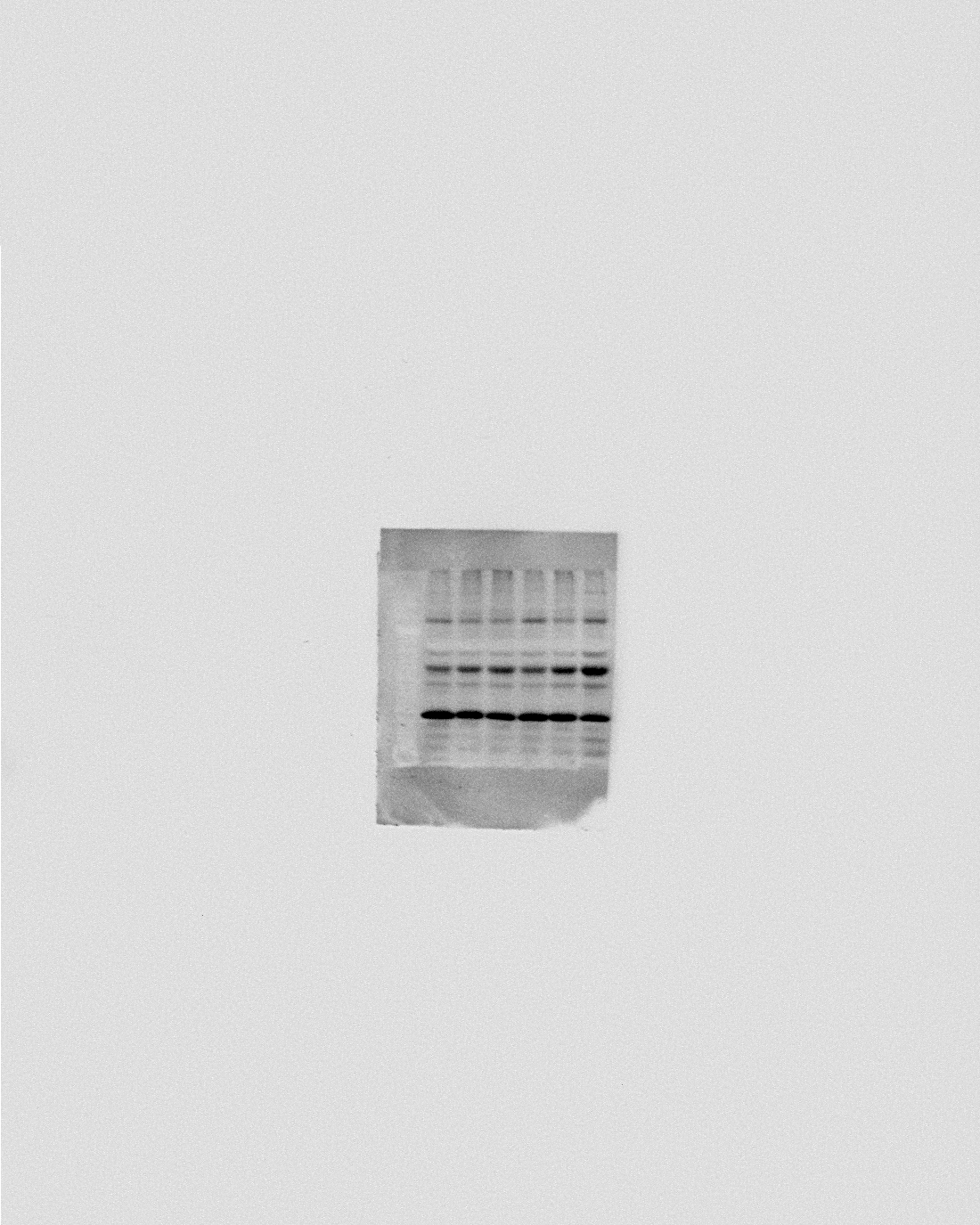

Supplement: S2 Data — (ZIP) [file pone.0256066.s002.zip › WB blots/WB Fig 7/Collagen ó≤-Fig 7C.tif]

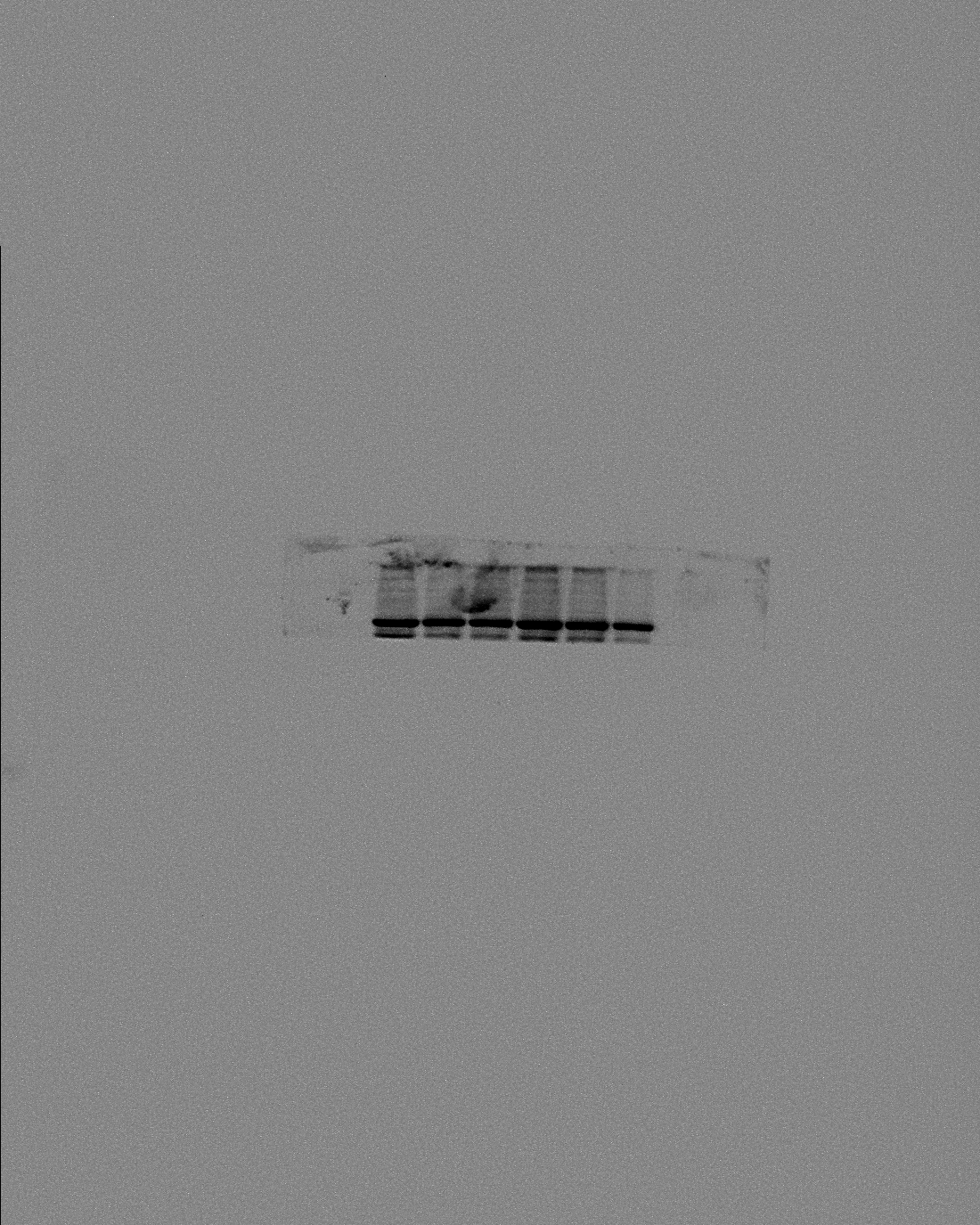

Supplement: S2 Data — (ZIP) [file pone.0256066.s002.zip › WB blots/WB Fig 7/nNOS-Fig 7A.tif]

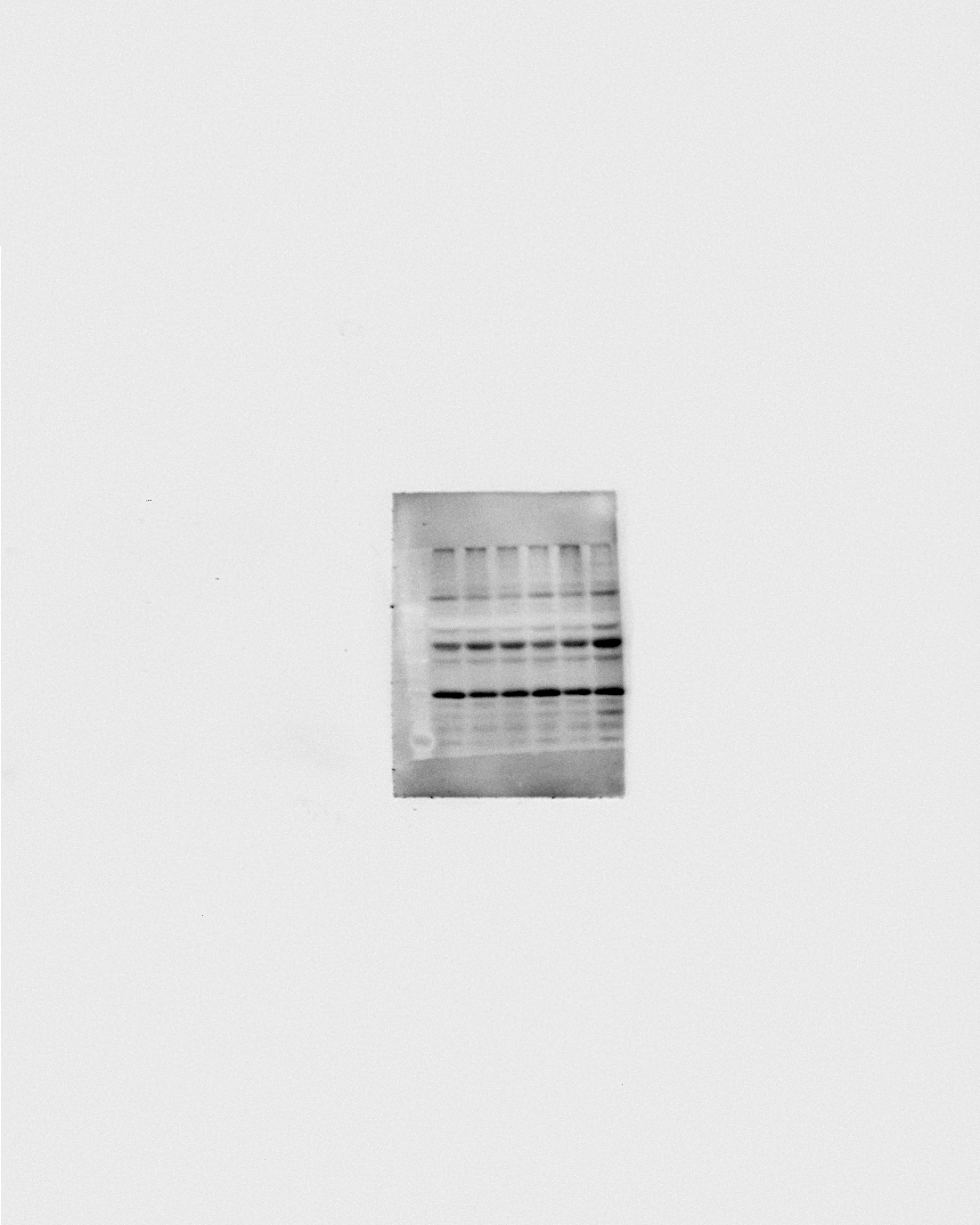

Supplement: S2 Data — (ZIP) [file pone.0256066.s002.zip › WB blots/WB Fig 7/a┴-SMA.tif]

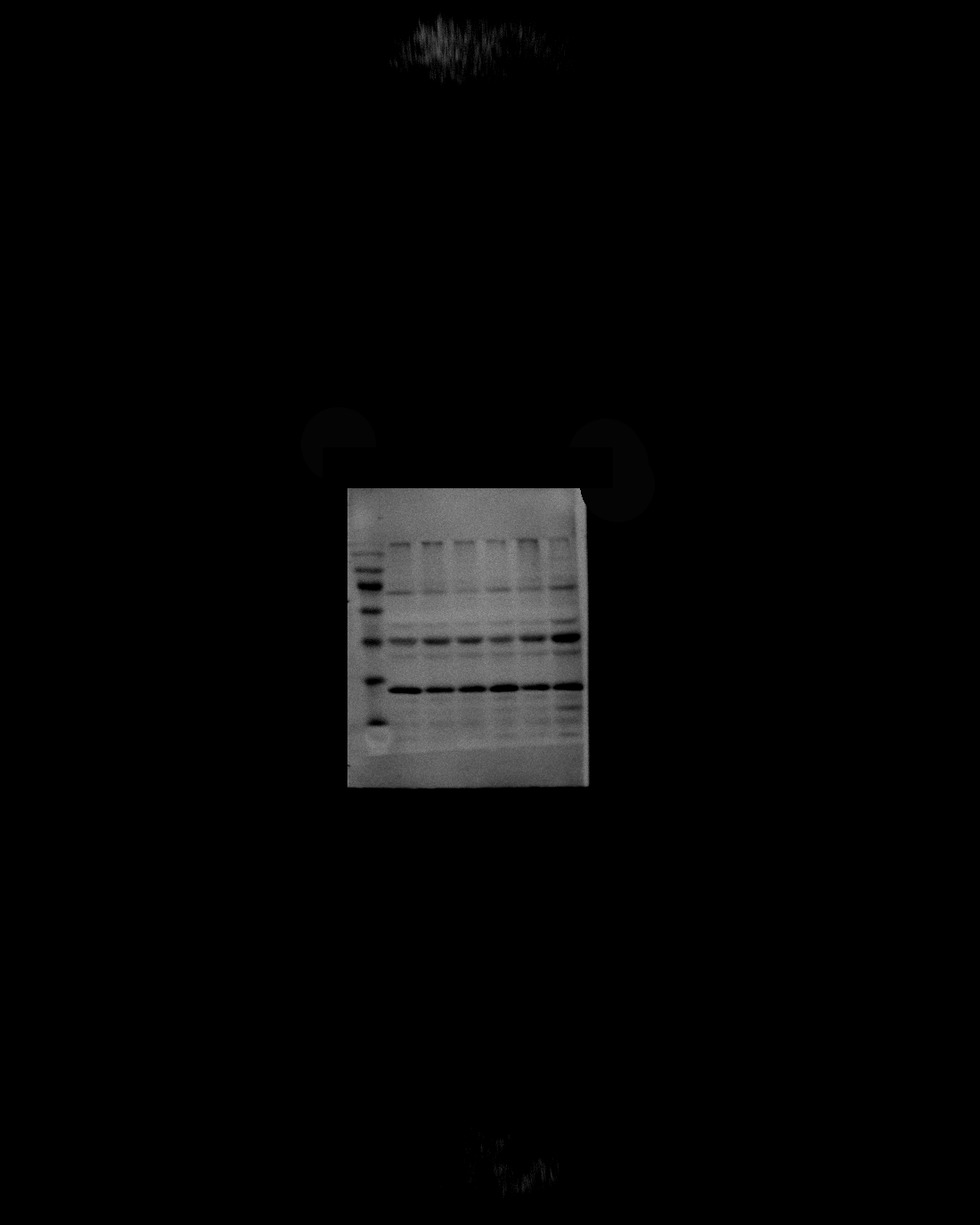

Supplement: S2 Data — (ZIP) [file pone.0256066.s002.zip › WB blots/WB Fig 7/a┴-SMA-Fig 7C.tif]

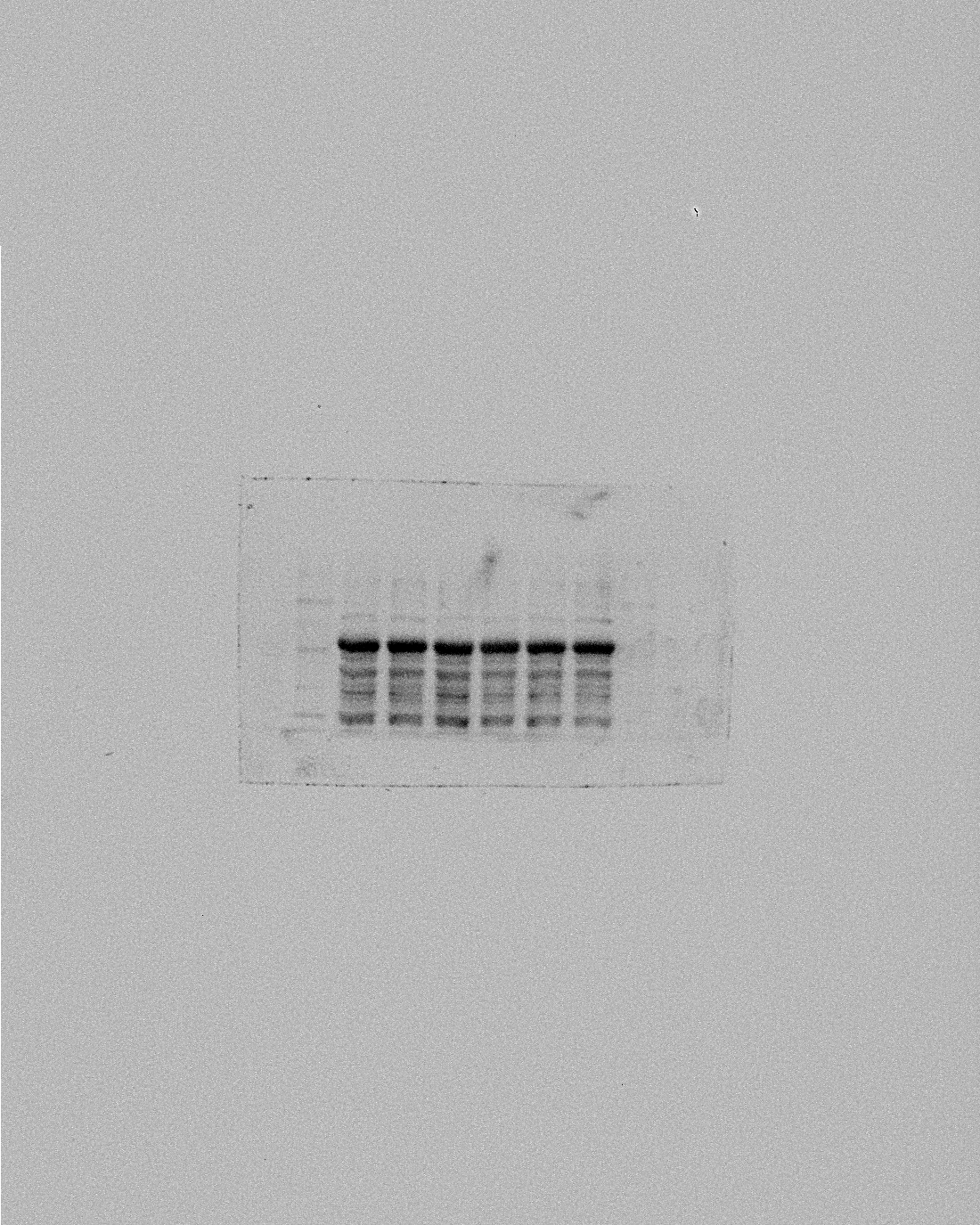

Supplement: S2 Data — (ZIP) [file pone.0256066.s002.zip › WB blots/WB Fig 8/actin.tif]

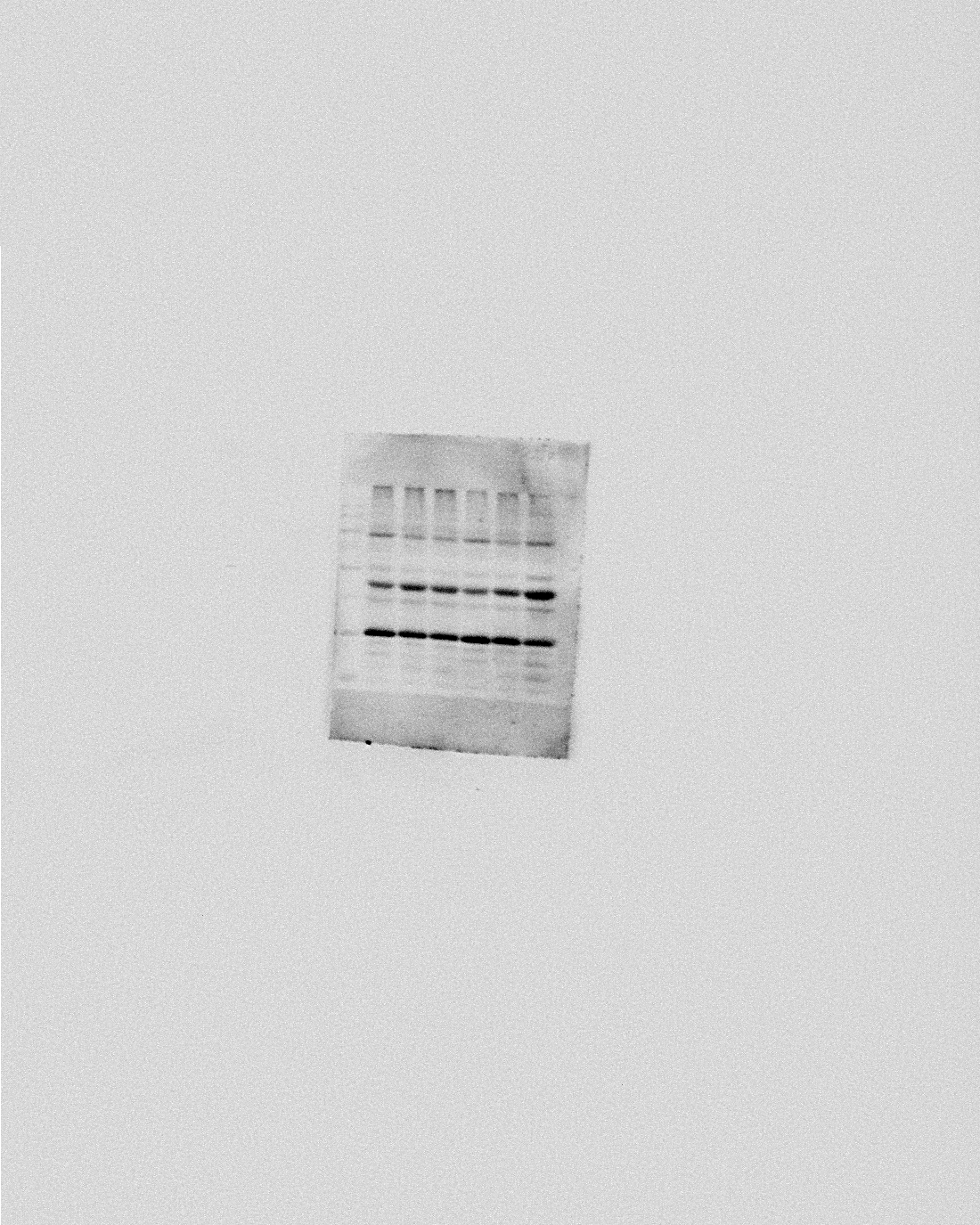

Supplement: S2 Data — (ZIP) [file pone.0256066.s002.zip › WB blots/WB Fig 8/p-smad23.tif]

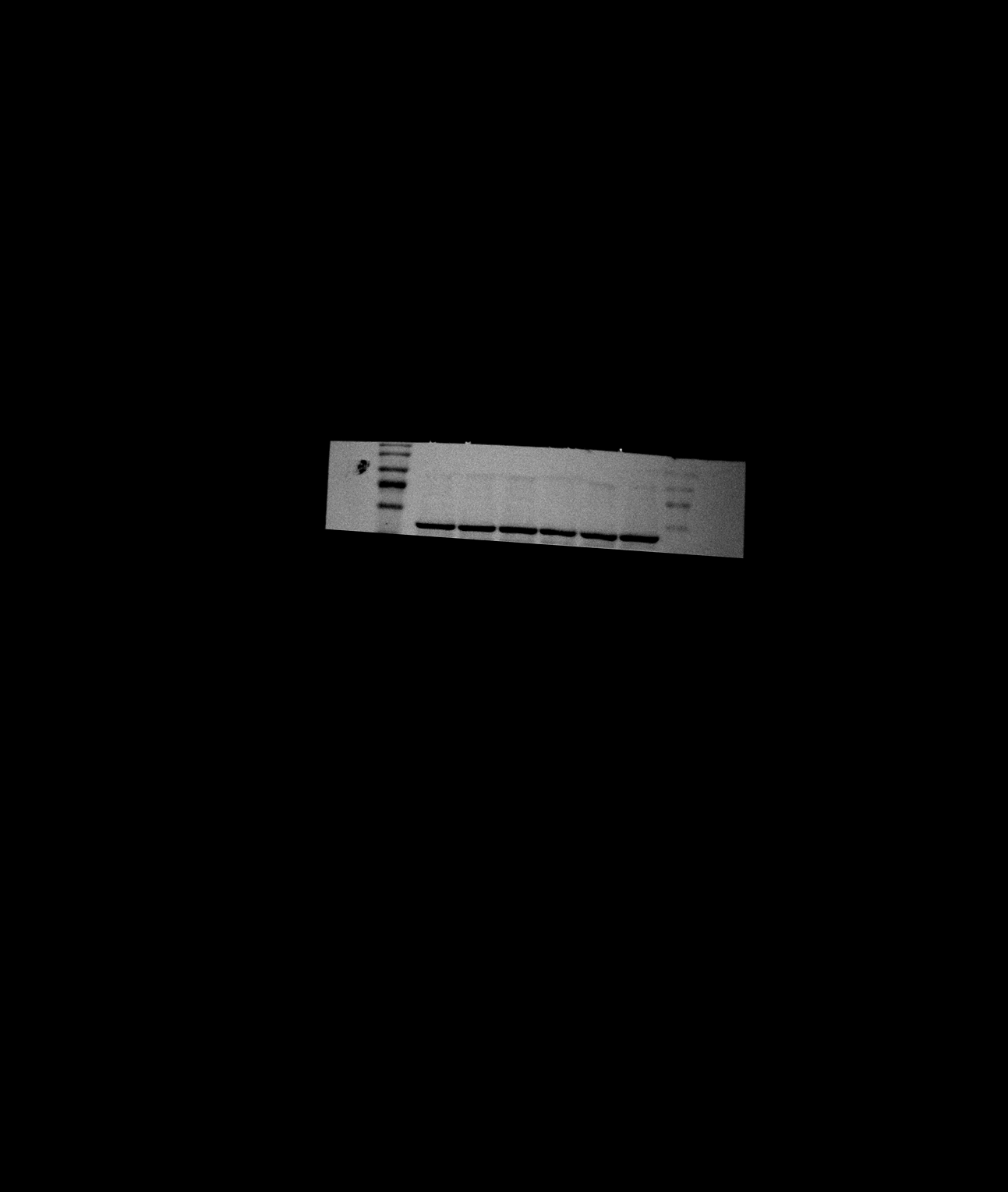

Supplement: S2 Data — (ZIP) [file pone.0256066.s002.zip › WB blots/WB Fig 8/smad23.tif]

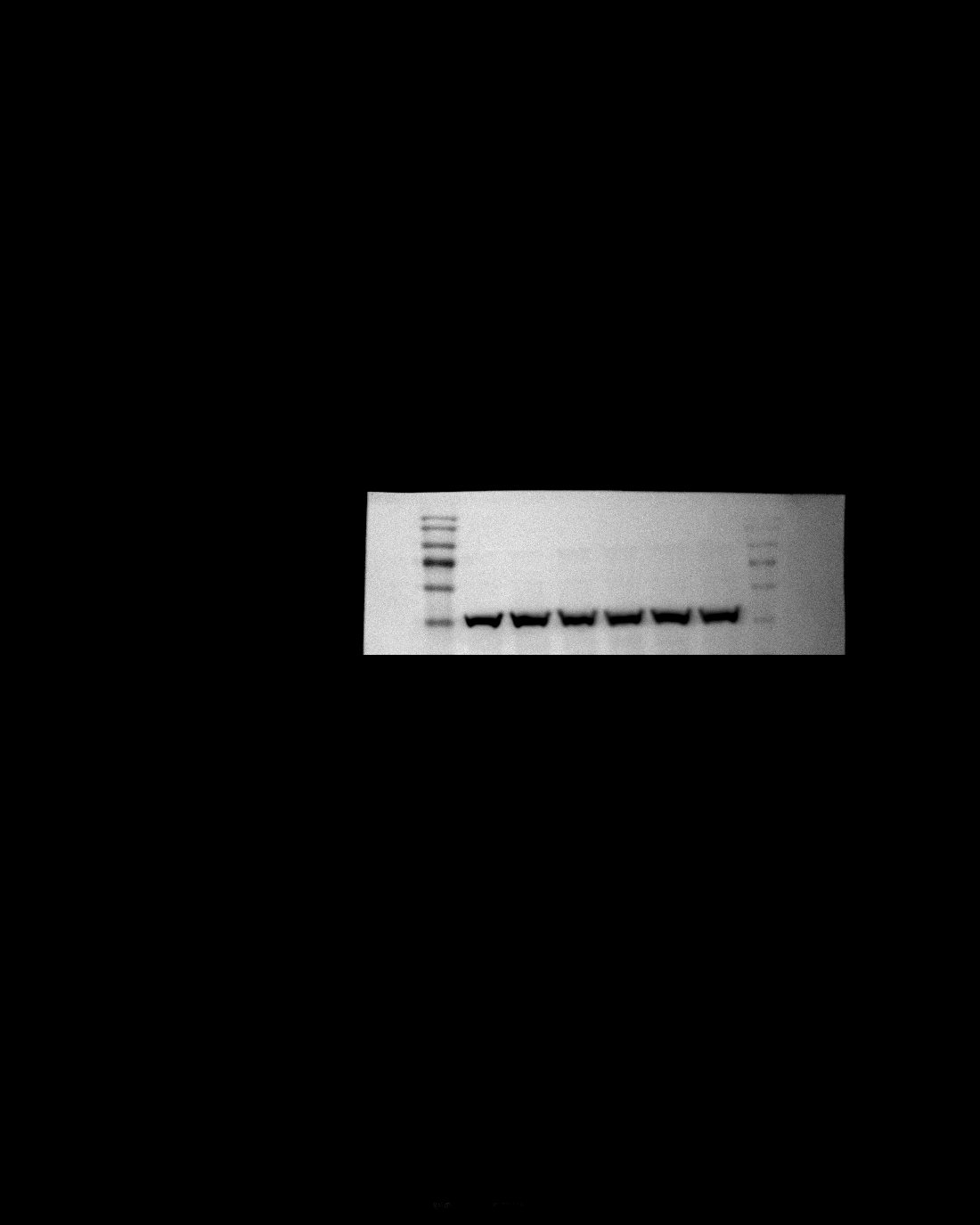

Supplement: S2 Data — (ZIP) [file pone.0256066.s002.zip › WB blots/WB Fig 9/actin-Fig 9A.tif]

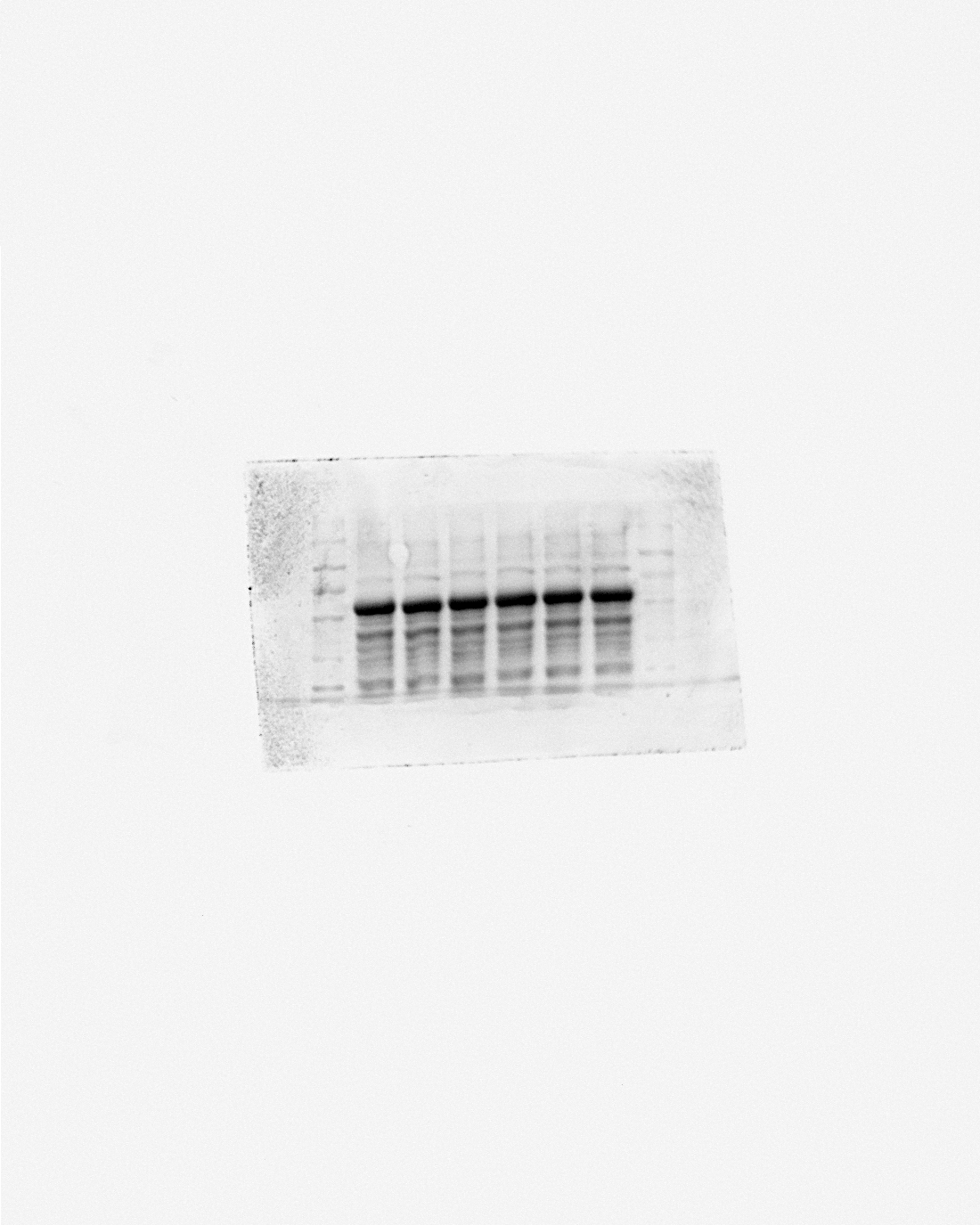

Supplement: S2 Data — (ZIP) [file pone.0256066.s002.zip › WB blots/WB Fig 9/actin-Fig 9C.tif]

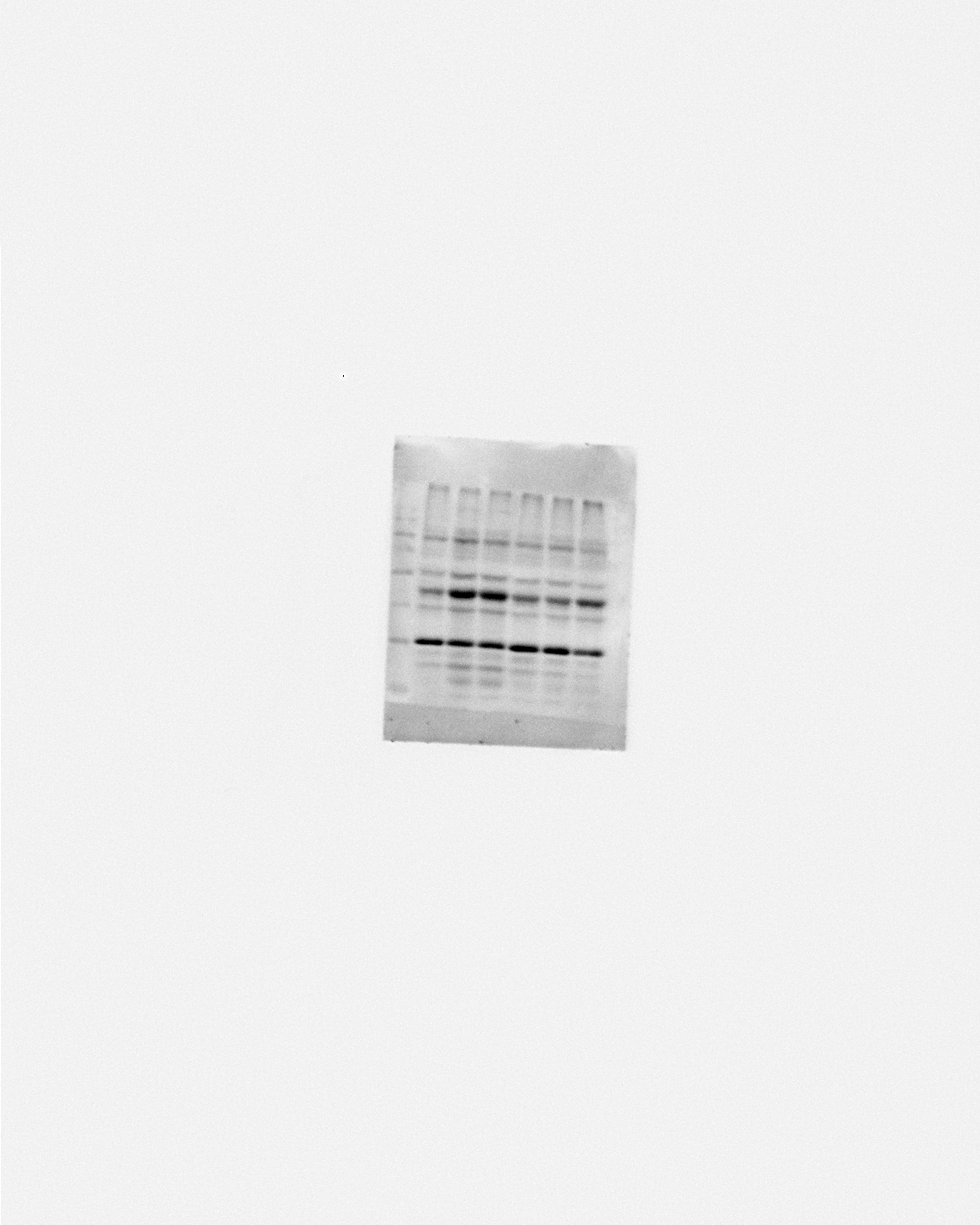

Supplement: S2 Data — (ZIP) [file pone.0256066.s002.zip › WB blots/WB Fig 9/Collagen ó±--Fig 9C.tif]

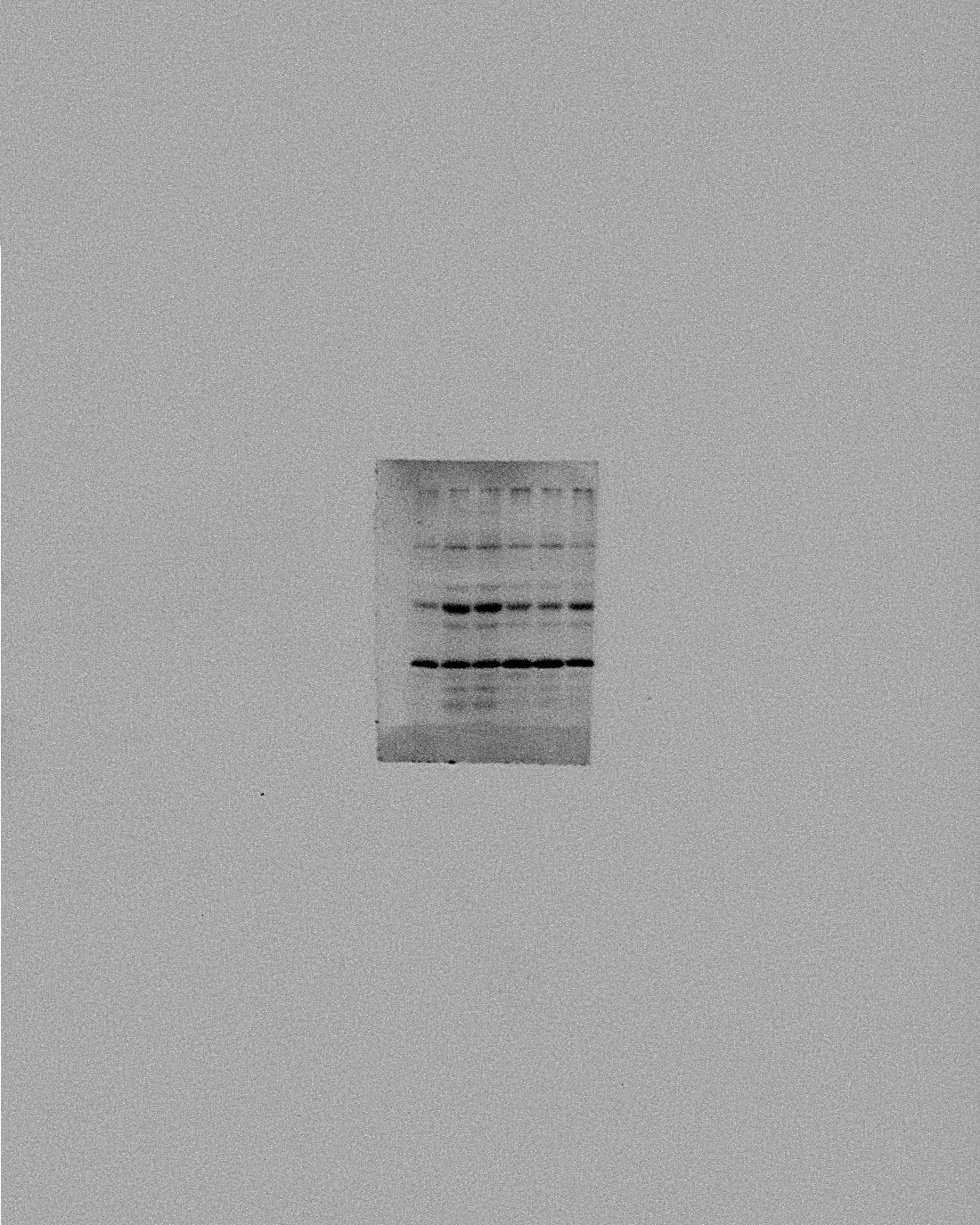

Supplement: S2 Data — (ZIP) [file pone.0256066.s002.zip › WB blots/WB Fig 9/Collagen ó≤-Fig 9C.tif]

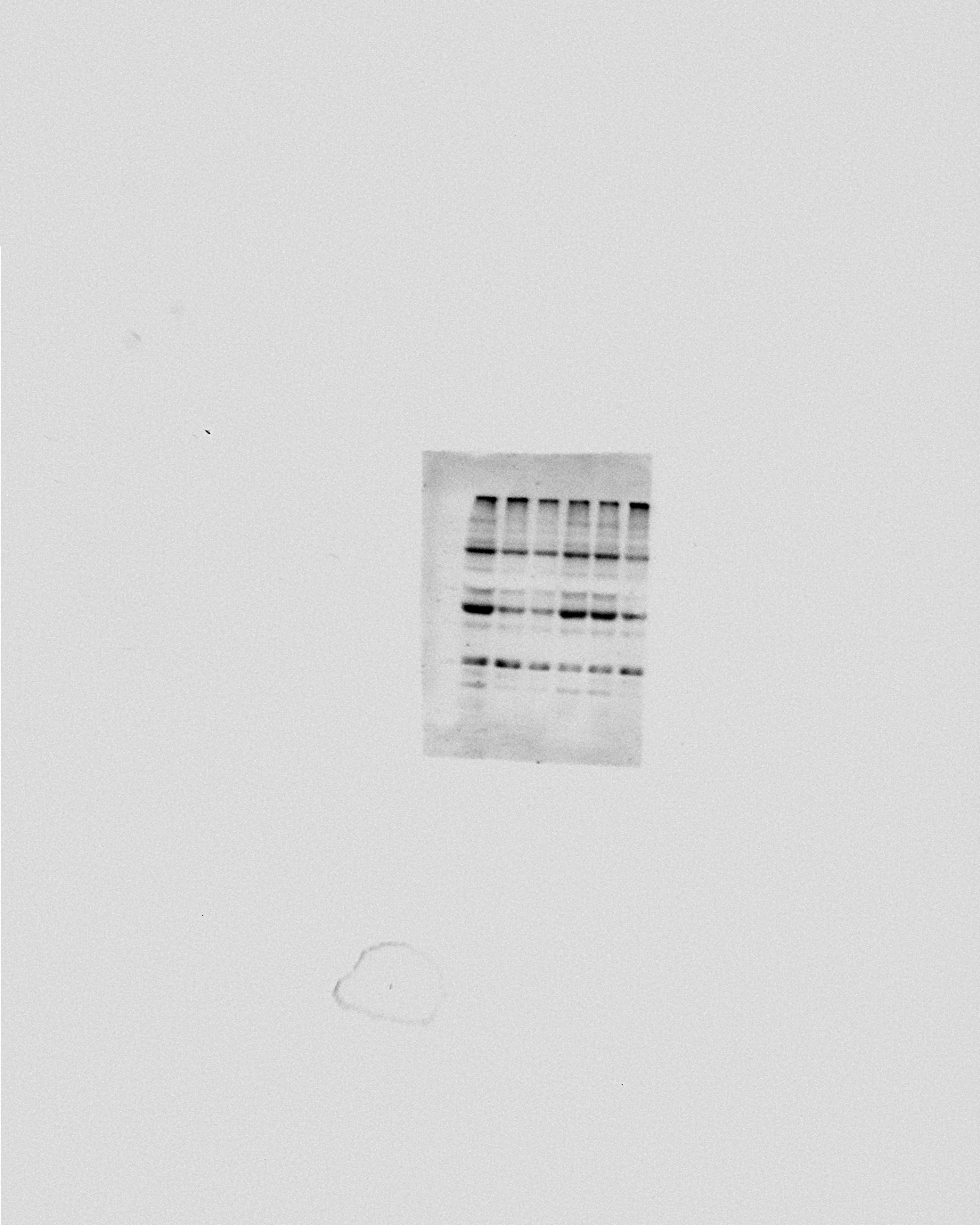

Supplement: S2 Data — (ZIP) [file pone.0256066.s002.zip › WB blots/WB Fig 9/nNOS-Fig 9A.tif]

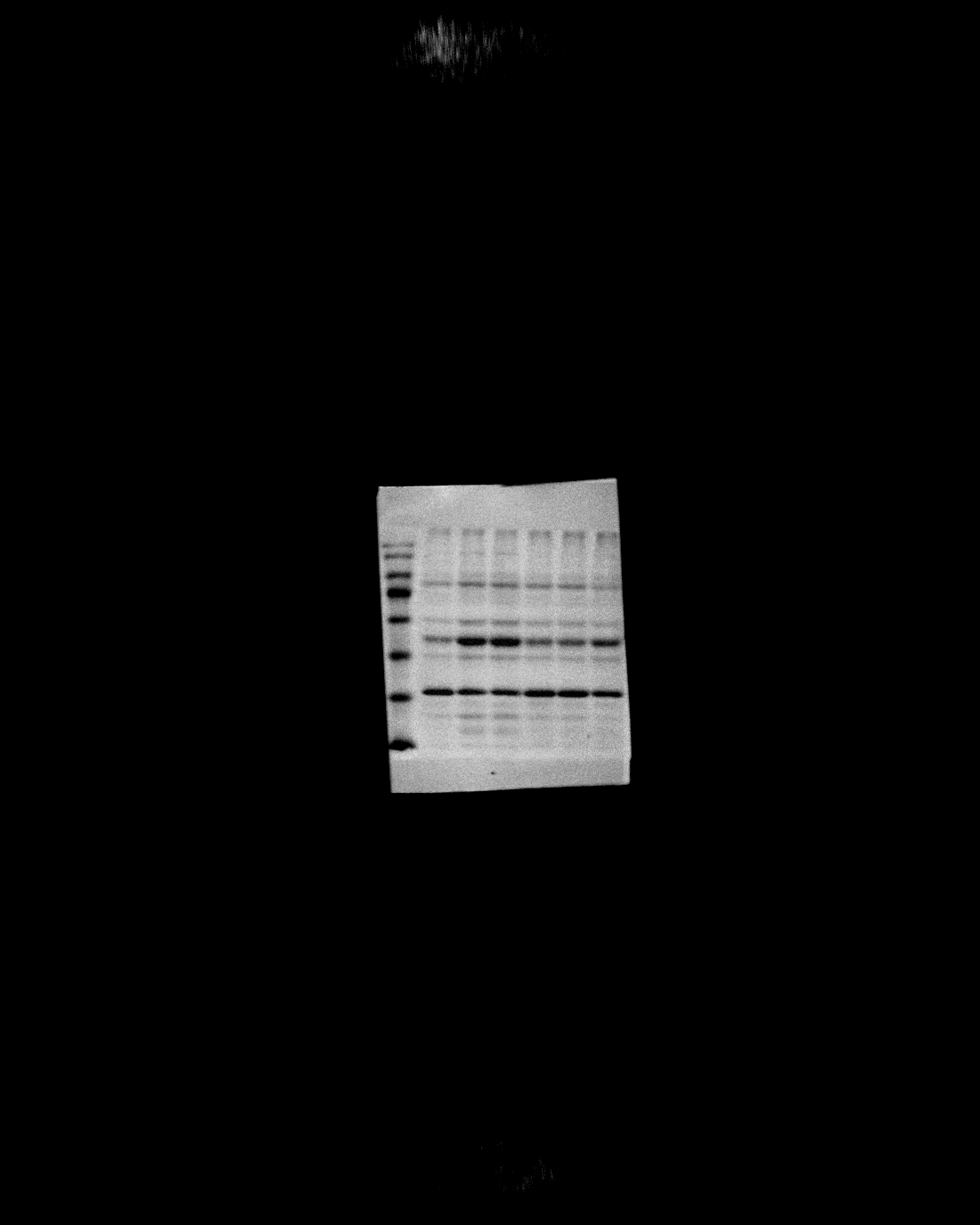

Supplement: S2 Data — (ZIP) [file pone.0256066.s002.zip › WB blots/WB Fig 9/a┴-SMA -Fig 9C.tif]

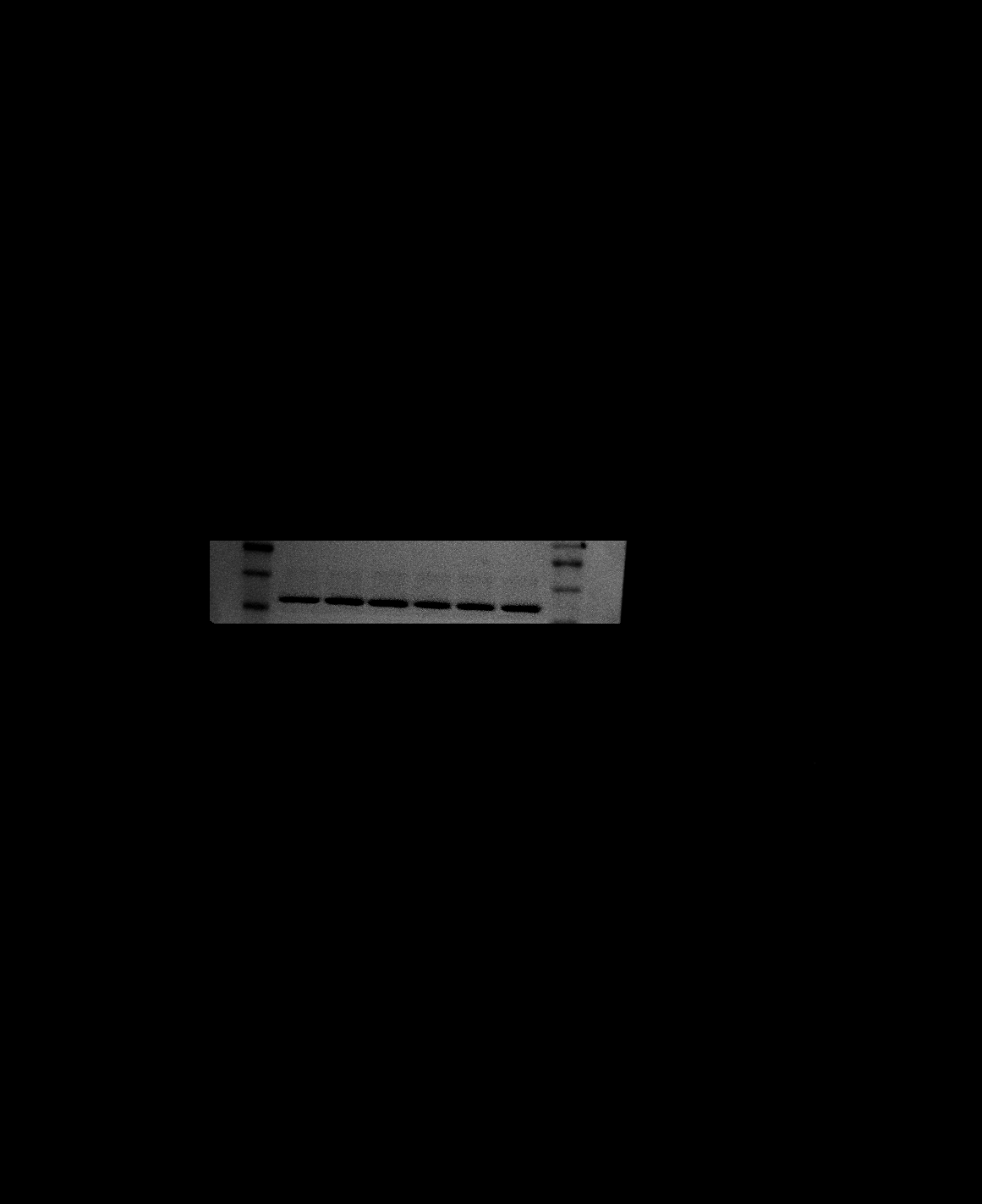

Supplement: S2 Data — (ZIP) [file pone.0256066.s002.zip › WB blots/WB-Fig 3/Fig 3A/actin.tif]

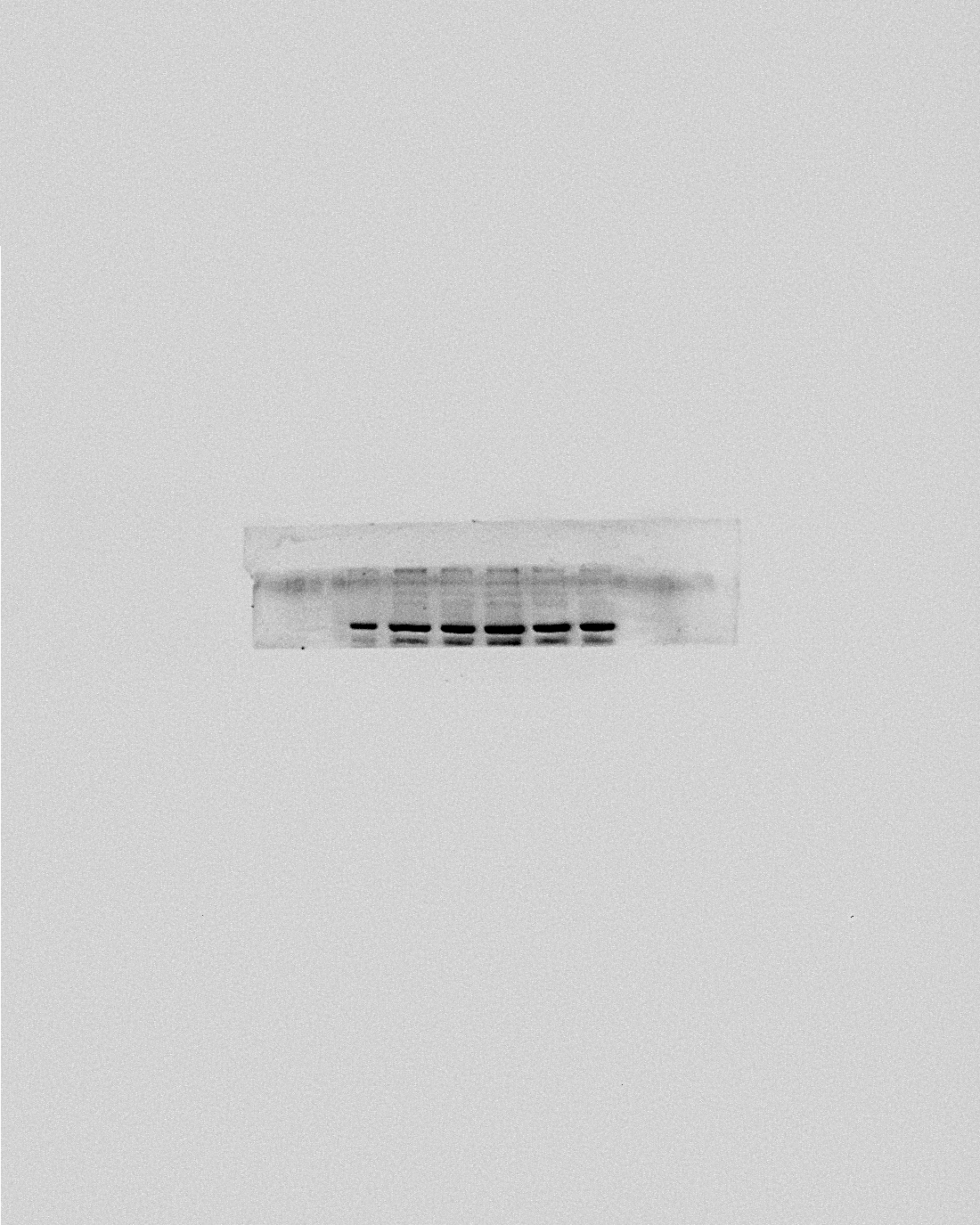

Supplement: S2 Data — (ZIP) [file pone.0256066.s002.zip › WB blots/WB-Fig 3/Fig 3A/Collagen ó≤.tif]

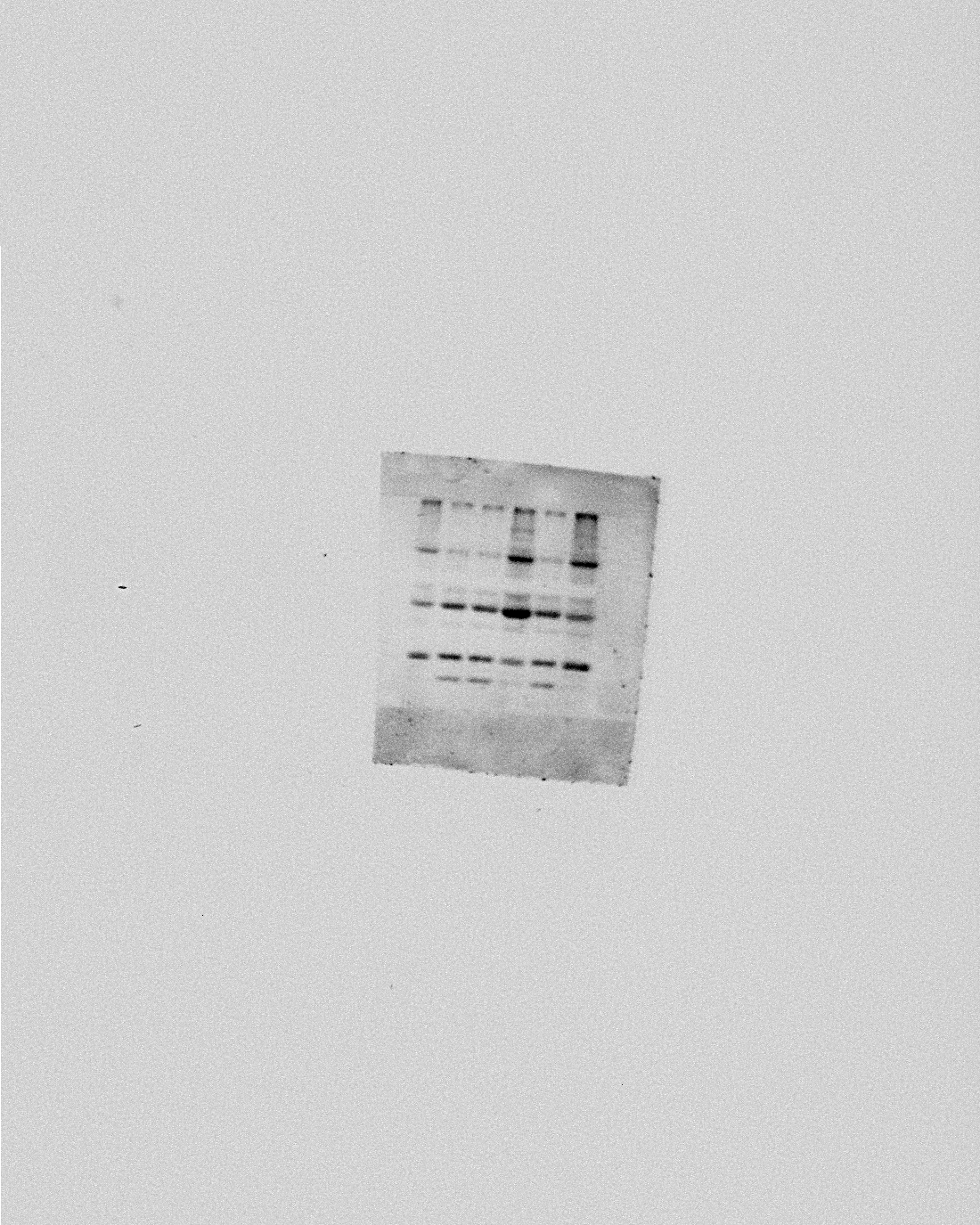

Supplement: S2 Data — (ZIP) [file pone.0256066.s002.zip › WB blots/WB-Fig 3/Fig 3A/Collagen I.tif]

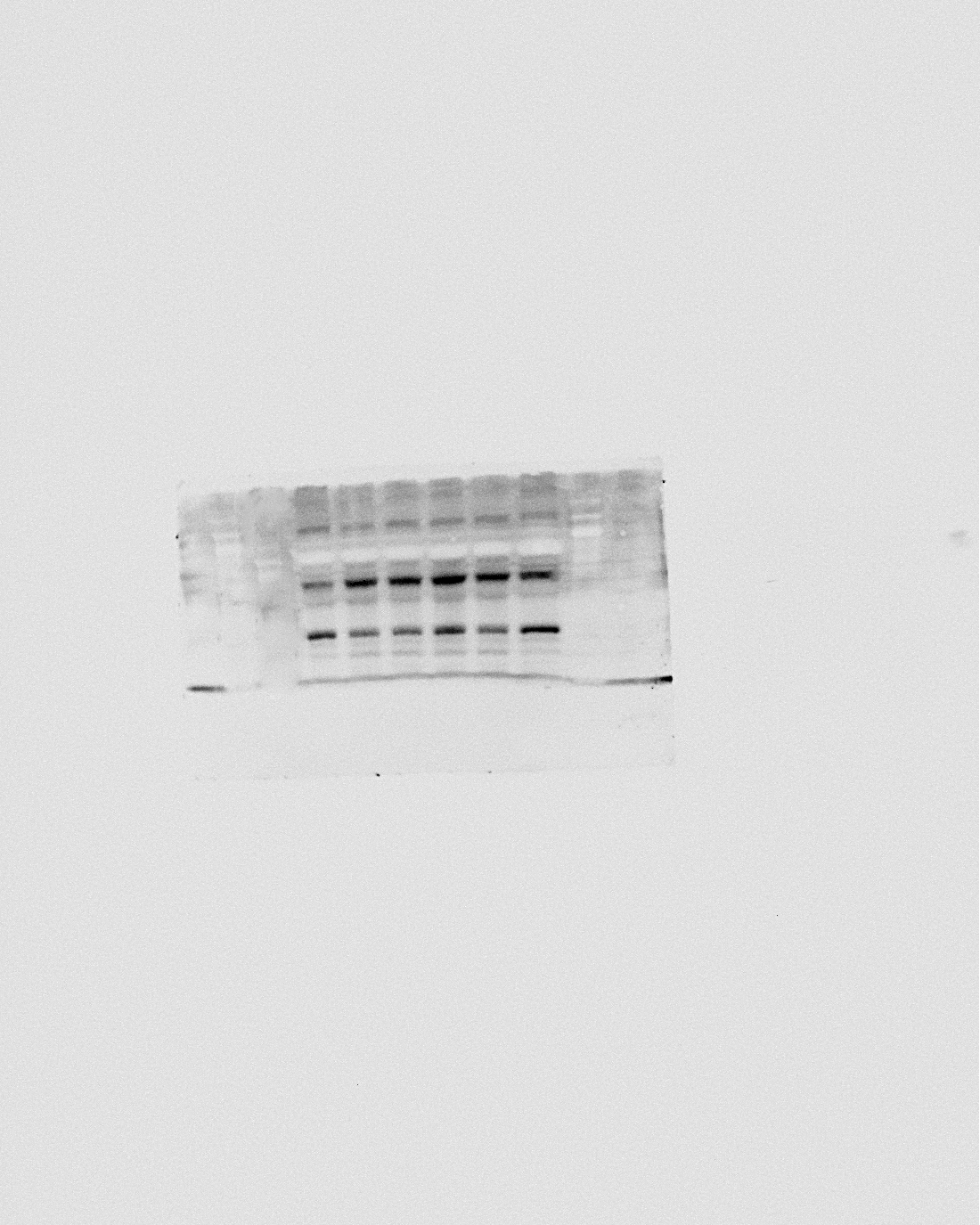

Supplement: S2 Data — (ZIP) [file pone.0256066.s002.zip › WB blots/WB-Fig 3/Fig 3A/a┴-SMA.tif]

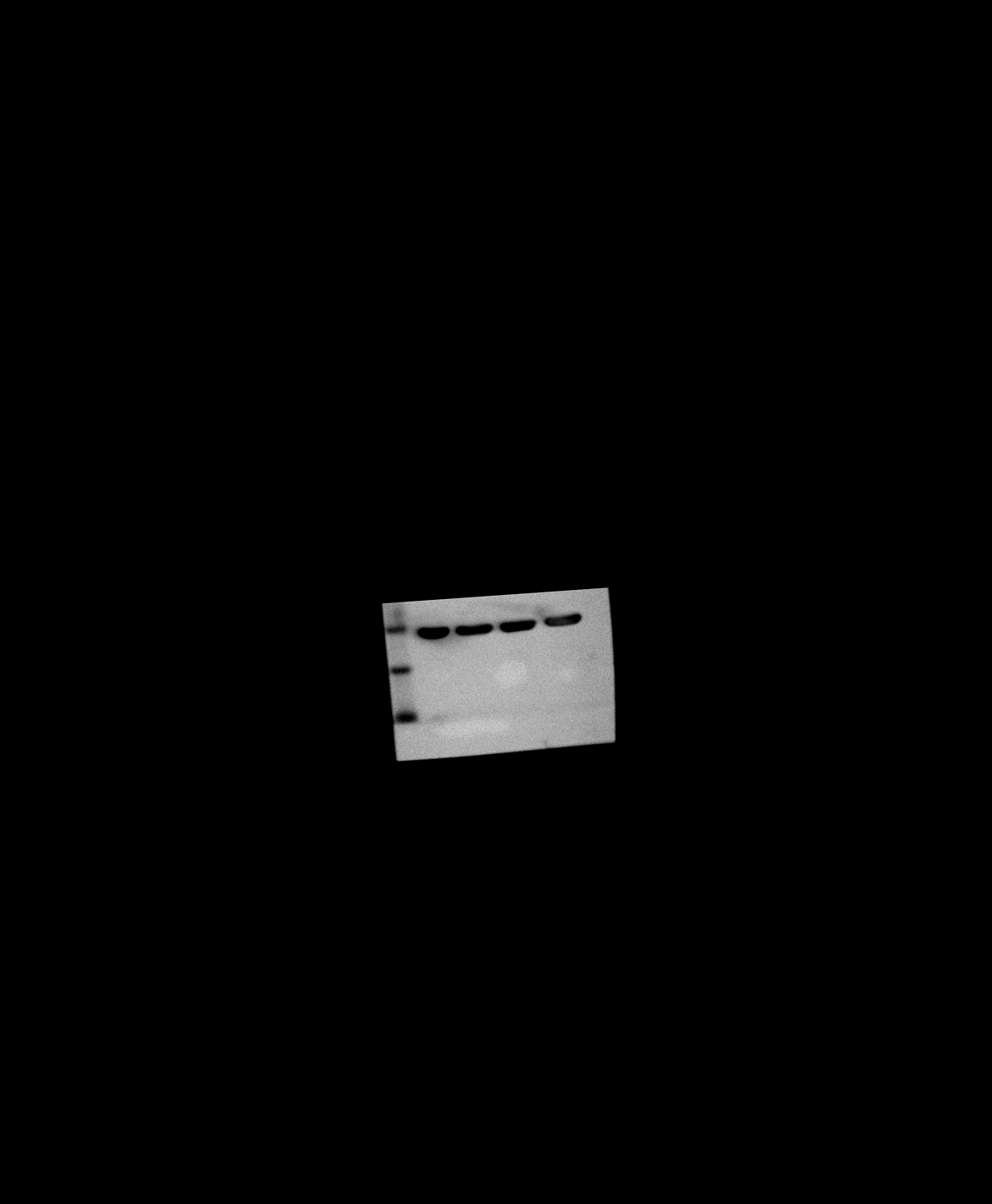

Supplement: S2 Data — (ZIP) [file pone.0256066.s002.zip › WB blots/WB-Fig 3/Fig 3B/actin.tif]

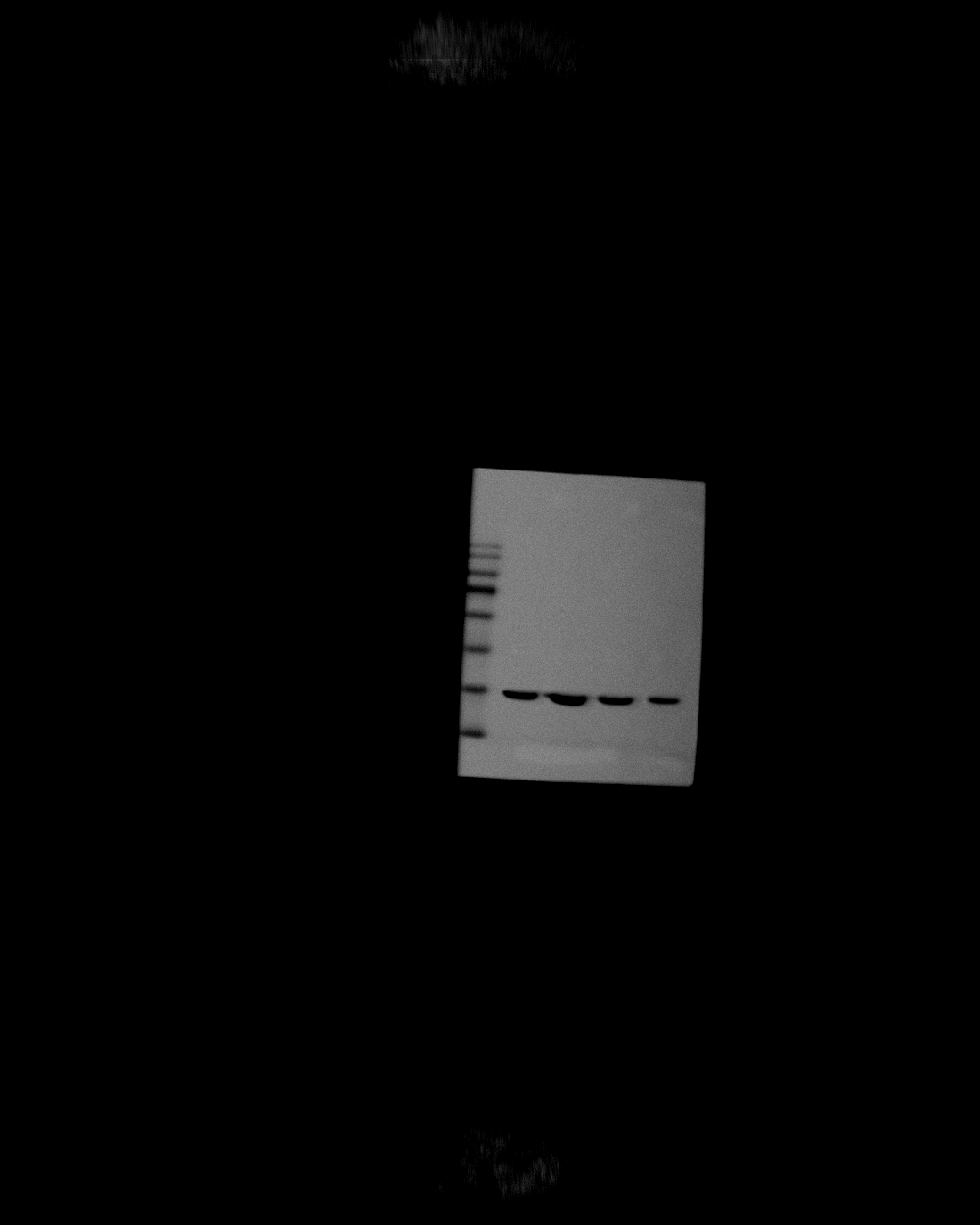

Supplement: S2 Data — (ZIP) [file pone.0256066.s002.zip › WB blots/WB-Fig 3/Fig 3B/Collagen I.tif]

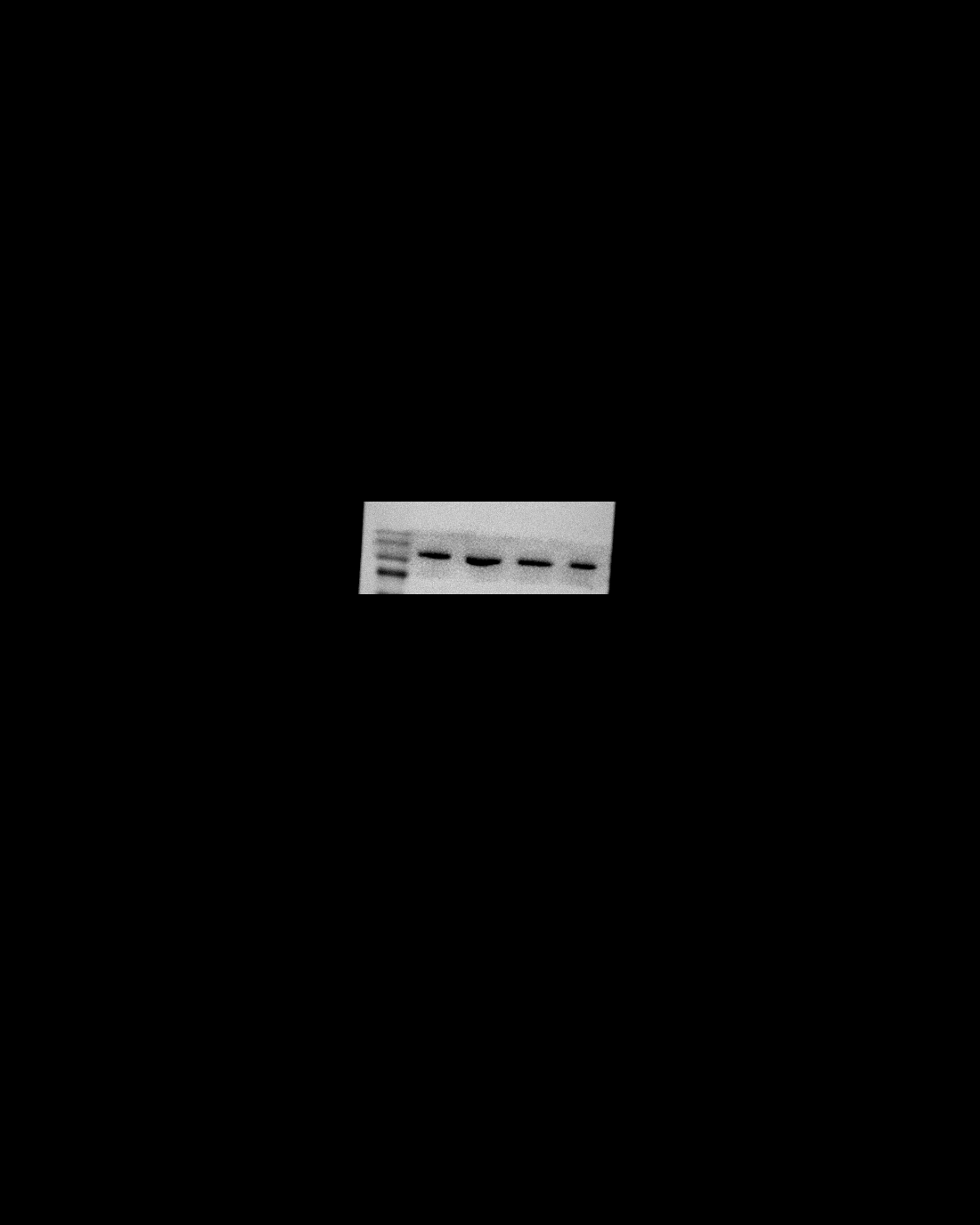

Supplement: S2 Data — (ZIP) [file pone.0256066.s002.zip › WB blots/WB-Fig 3/Fig 3B/Collagen III.tif]

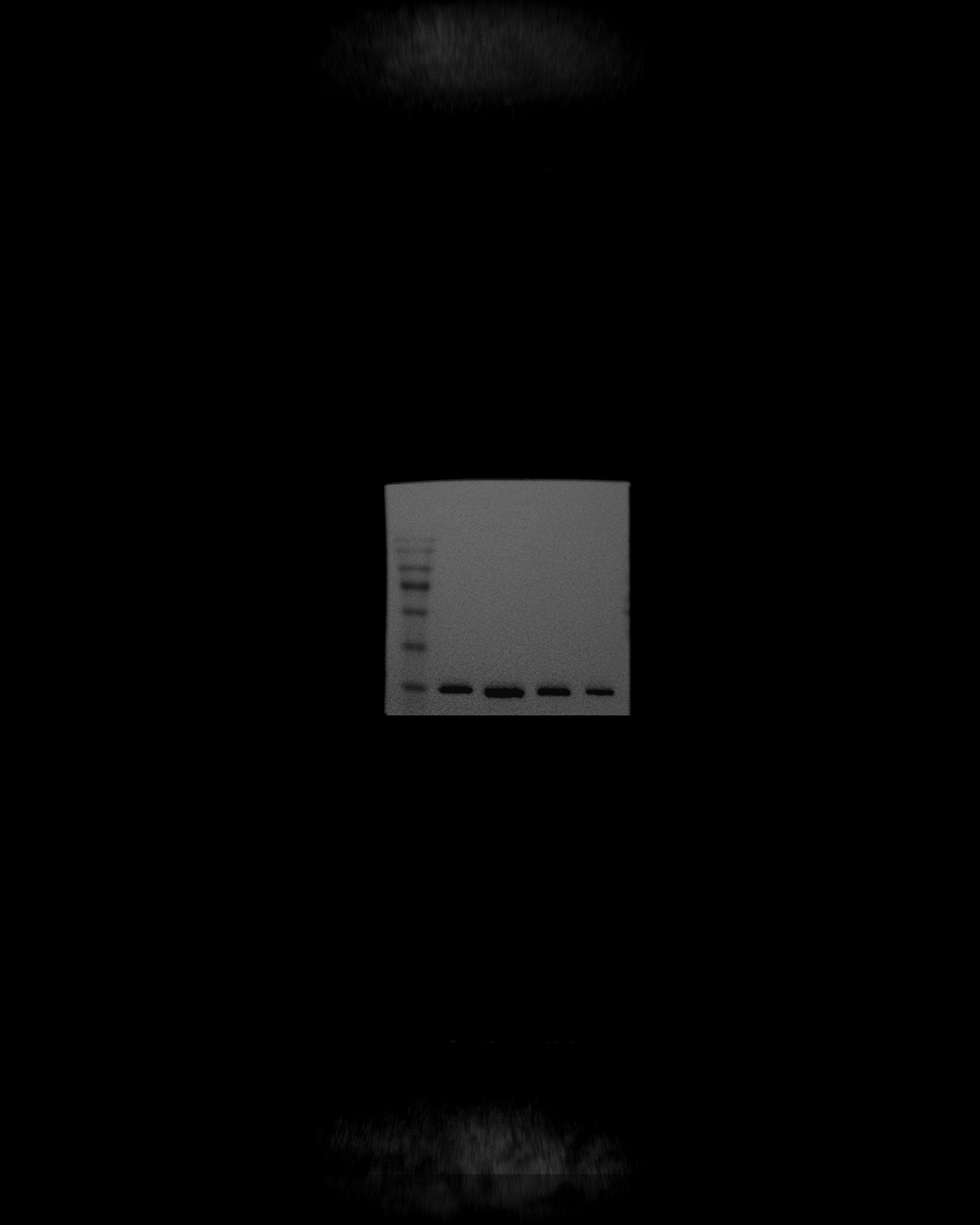

Supplement: S2 Data — (ZIP) [file pone.0256066.s002.zip › WB blots/WB-Fig 3/Fig 3B/a┴-SMA.tif]

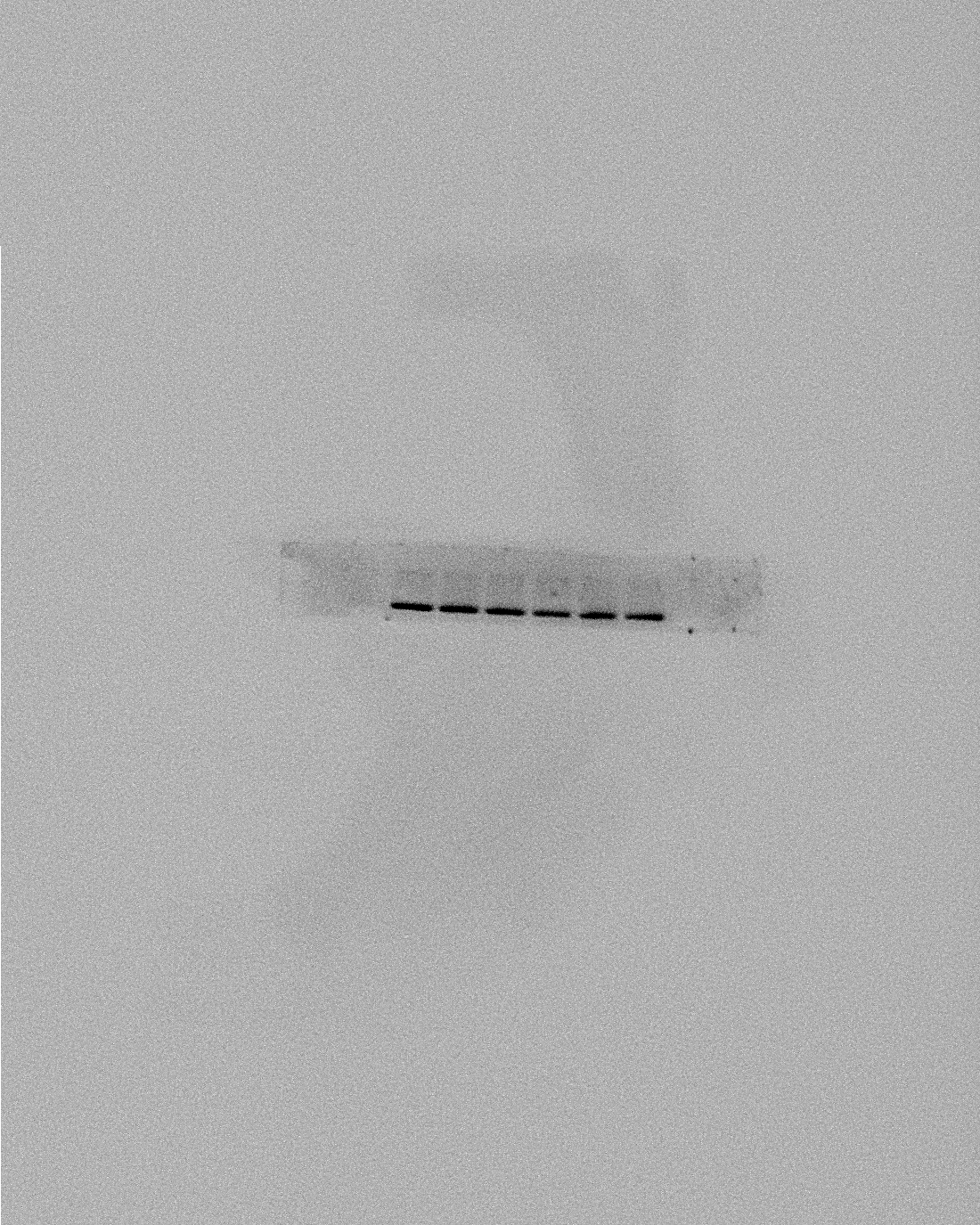

Supplement: S2 Data — (ZIP) [file pone.0256066.s002.zip › WB blots/WB-Fig 5/actin.tif]

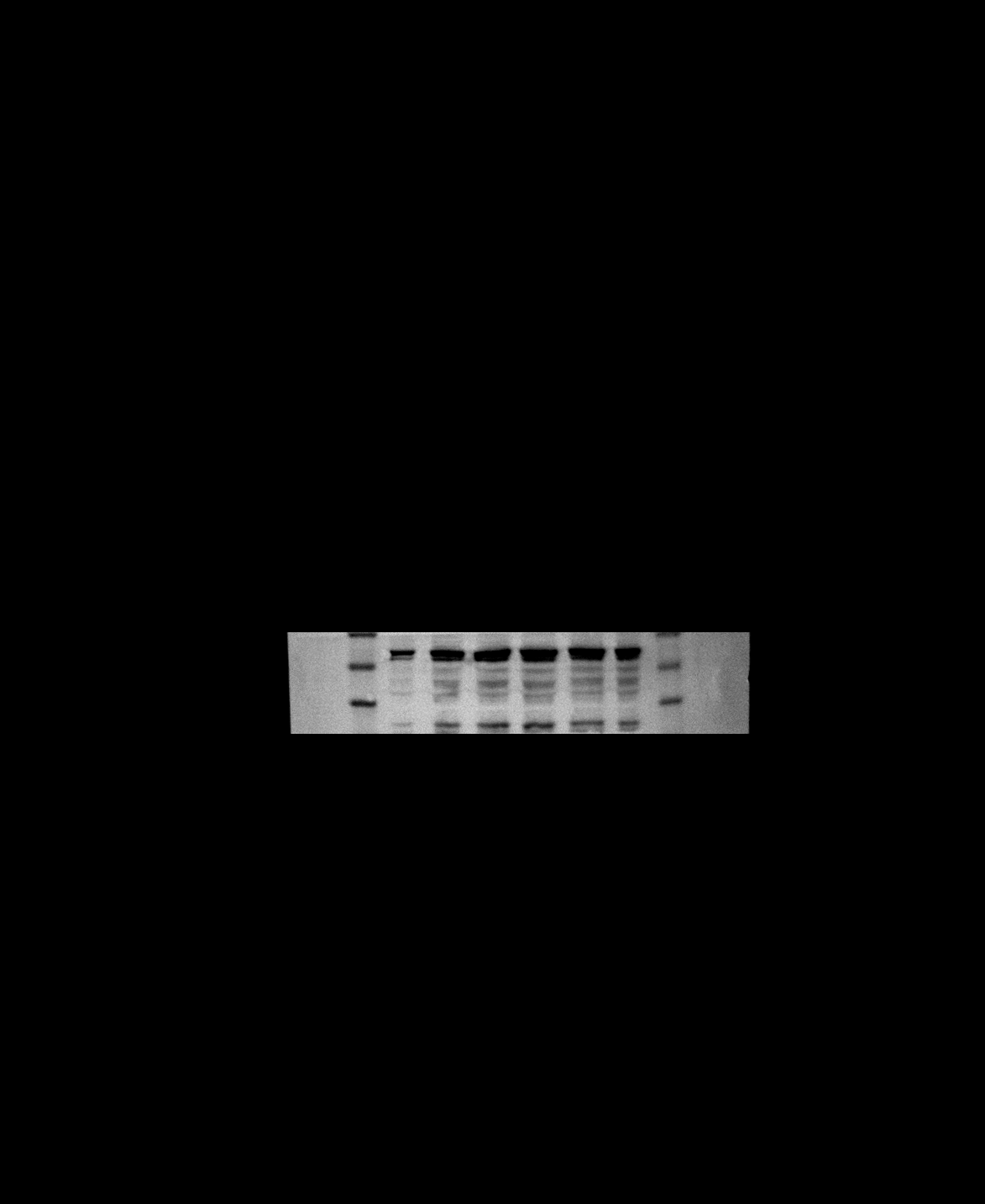

Supplement: S2 Data — (ZIP) [file pone.0256066.s002.zip › WB blots/WB-Fig 5/p-Smad23.tif]

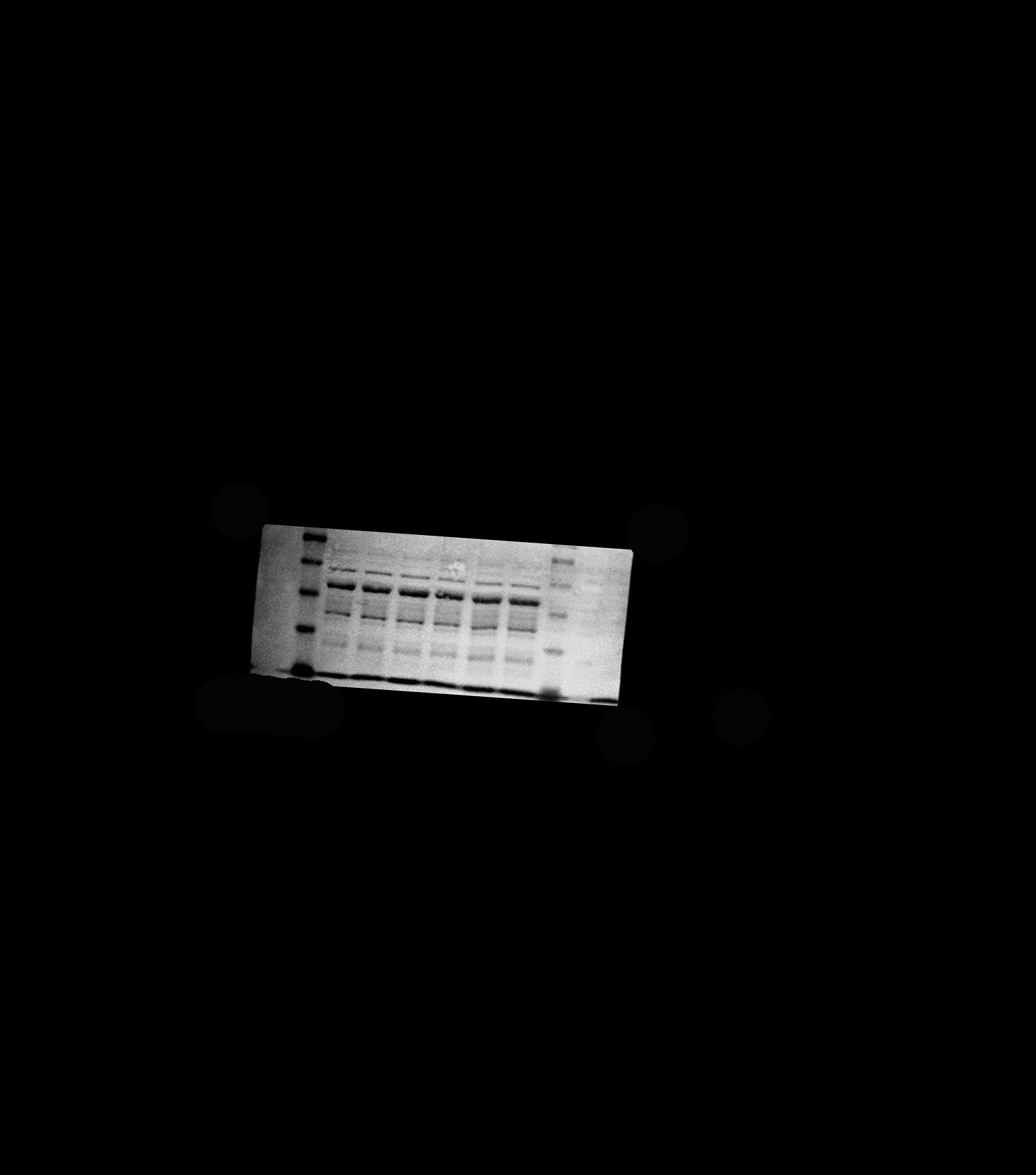

Supplement: S2 Data — (ZIP) [file pone.0256066.s002.zip › WB blots/WB-Fig 5/Smad23.tif]

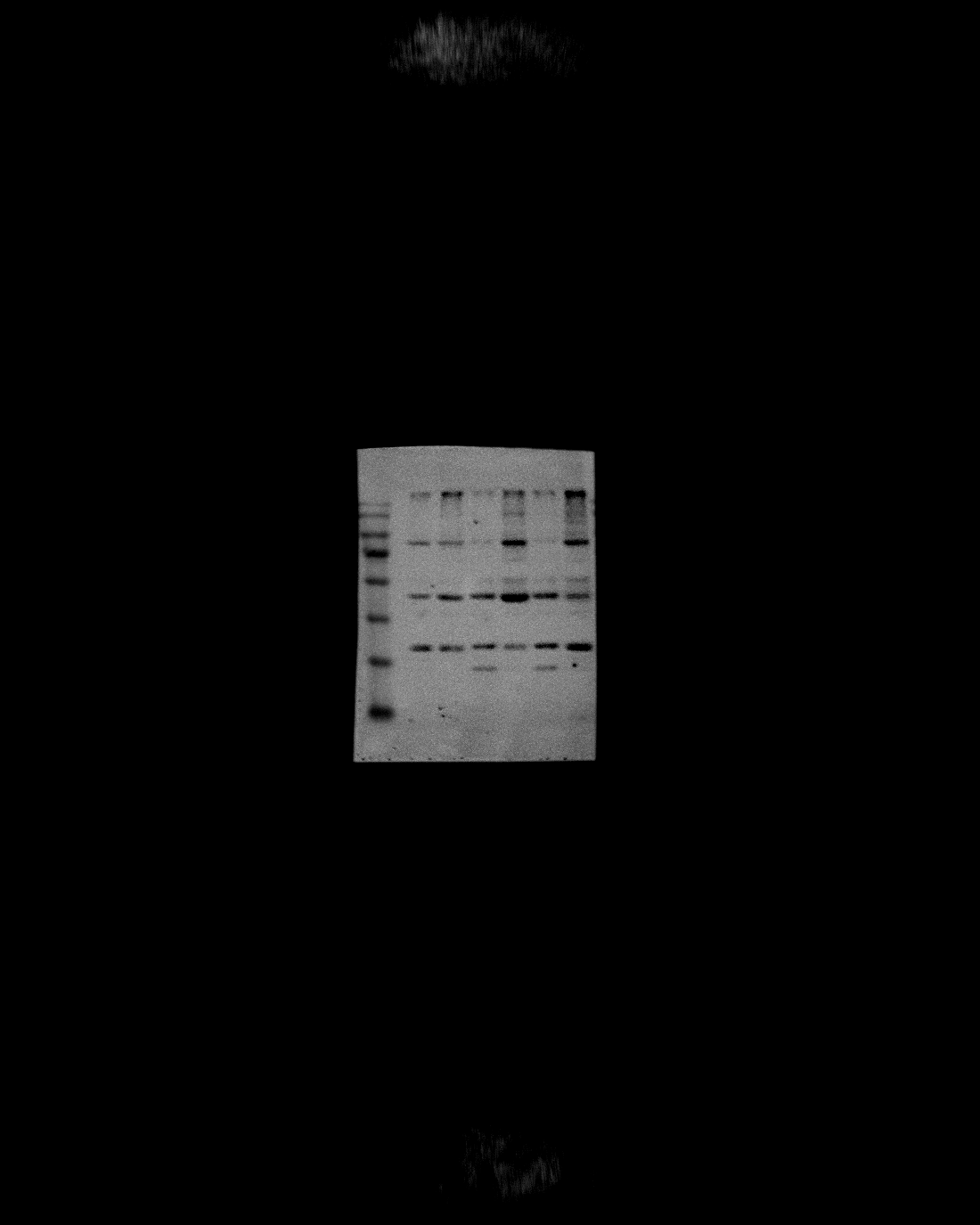

Supplement: S2 Data — (ZIP) [file pone.0256066.s002.zip › WB blots/WB-Fig 5/TGF-a┬.tif]
